# Molecular mechanosensors in osteocytes

**DOI:** 10.1038/s41413-020-0099-y

**Published:** 2020-06-08

**Authors:** Lei Qin, Wen Liu, Huiling Cao, Guozhi Xiao

**Affiliations:** Guangdong Provincial Key Laboratory of Cell Microenvironment and Disease Research, Shenzhen Key Laboratory of Cell Microenvironment, and School of Medicine, Southern University of Science and Technology, Shenzhen, 518055 China

**Keywords:** Bone quality and biomechanics, Osteoporosis

## Abstract

Osteocytes, the most abundant and long-lived cells in bone, are the master regulators of bone remodeling. In addition to their functions in endocrine regulation and calcium and phosphate metabolism, osteocytes are the major responsive cells in force adaptation due to mechanical stimulation. Mechanically induced bone formation and adaptation, disuse-induced bone loss and skeletal fragility are mediated by osteocytes, which sense local mechanical cues and respond to these cues in both direct and indirect ways. The mechanotransduction process in osteocytes is a complex but exquisite regulatory process between cells and their environment, between neighboring cells, and between different functional mechanosensors in individual cells. Over the past two decades, great efforts have focused on finding various mechanosensors in osteocytes that transmit extracellular mechanical signals into osteocytes and regulate responsive gene expression. The osteocyte cytoskeleton, dendritic processes, Integrin-based focal adhesions, connexin-based intercellular junctions, primary cilium, ion channels, and extracellular matrix are the major mechanosensors in osteocytes reported so far with evidence from both in vitro and in vitro studies. This review aims to give a systematic introduction to osteocyte mechanobiology, provide details of osteocyte mechanosensors, and discuss the roles of osteocyte mechanosensitive signaling pathways in the regulation of bone homeostasis.

## Introduction

Osteocytes are the most abundant and long-lived cell type in bone, accounting for 90%–95% of total bone cells in the adult skeleton.^1^ Although osteocytes are terminally differentiated cells derived from osteoblasts, bone contains ten times more osteocytes than osteoblasts.^2^ Over the last two to three decades, osteocytes, previously seen as a “passive placeholder” in mineralized bone, have emerged as a new multifunctional “superstar” in bone research.^1^ First, osteocytes are the master regulator of bone homeostasis through their direct regulation of local calcium abundance in mineralization and indirect control of osteoblast (bone-forming cell) and osteoclast (bone-resorbing cell) activities by the secretion of important regulatory factors.^3–5^ Second, osteocytes are endocrine cells that regulate phosphate metabolism in multiple organs, such as the kidney and parathyroid.^1,6–8^ Last, but the most importantly, osteocytes function as the principal regulators of bone mechanosensation and mechanotransduction.^1,9–11^

Mechanical stimuli induce and regulate various cellular functions, such as gene expression, protein synthesis, cell proliferation, and differentiation.^12,13^ Galileo was a pioneer who observed and described that in bone tissue “loading is required to preserve bone mass.”^10^ In 1892, the German surgeon Julius Wolff introduced his famous “Wolff’s Law,” stating that bone growth and remodeling occur in response to forces placed upon bone in a healthy person.^10,14^ In the 1980s, Harold Frost was the first to use the word “mechanostat” to describe the mechanism underlying this load-induced bone adaptation process and identify osteocytes as the “mechanostat” of bone.^10,15^

During mechanical stimulation from daily activities, whole-body mechanics are transduced to the organ level, tissue level, and finally, cellular level.^16^ In bone tissue, osteocytes have been suggested to be the main cell type responsive to mechanical stimulation.^1,10,16^ Direct evidence for the mechanosensitive function of osteocytes was revealed in a study showing that transgenic mice with specific osteocyte ablation failed to respond to unloading-induced bone loss.^17^ The mechanical environment in the mineralized extracellular matrix (ECM), in which osteocytes are embedded, presents a dynamic combination of various biophysical stimuli, including strain, stress, shear, osmotic pressure, fluid flow, streaming potentials, and acceleration.^18^ Among these stimuli, the shear stress of fluid flow from loading is the main force stimulation applied to osteocytes.^9,16^

**Fig. 1:**
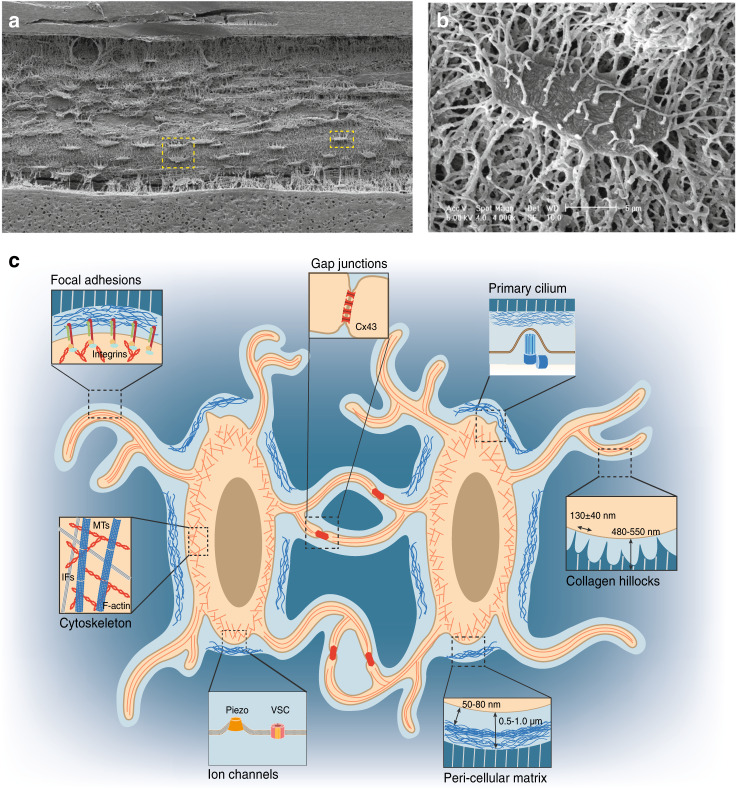
Osteocytes in the LCS of the bone environment. **a** SEM image of acid-etched resin-embedded cortical bone sections reveals an ellipsoid cell shape and extensive canaliculi connections among osteocytes.^8^**b** Magnified SEM image of a single osteocyte highlighted in the yellow square in **a**. **c** Illustration of osteocytes in the LCS of the bone environment. Magnified cartoon image of two adjacent osteocytes highlighted in the yellow square in **a**. The important aspects of osteocytes are highlighted in magnified cartoon images: focal adhesions, gap junctions, the primary cilium, cell cytoskeleton, ion channels, pericellular matrix at the lacunar region, and collagen hillocks at the canalicular region. [Panels **a** and **b** from Bonewald et al.,^8^ reprinted with permission]

The essential role of shear stress in osteocytes is determined by the natural physical environment of these cells, with osteocytes embedded in a lacuno-canalicular system (LCS) (Fig. 1). Transmission electron microscopy (TEM) analysis of fine murine bone sections revealed an average distance of 0.7 μm (0.1–2.0 μm) in the osteocyte lacuna, the space between the osteocyte cell body and mineralized ECM.^19^ A layer of collagen fibrils called the pericellular matrix (PCM), which is distinct from mineralized ECM, surrounds the osteocyte cell body in the lacuna. The PCM has a thickness of 0.5–1.0 μm and does not directly interact with the osteocyte cell surface, leaving a 50–80-nm space between cells and the PCM.^20^ In the osteocyte canaliculi, the canalicular diameter ranges from 210–260 nm.^21,22^ Moreover, collagen matrix projections from mineralized substrate form “hill-like” structures in osteocyte canaliculi that directly link the matrix and osteocyte dendrites. These structures are called “collagen hillocks”^20^ or “canalicular projections,”^22^ and an average internal space of 130 ± 40 nm exists between two projections.^20^ At the interface between collagen hillocks and osteocyte dendrites, Integrin-mediated focal adhesions (FAs) link the cell membrane and matrix^23^ and further transmit physical signals to the osteocyte cytoskeleton.

With their special LCS and extensive cell–matrix connections, osteocytes can sense shear stress from mechanical stimulation. A computational model focused on theoretical strain through the sliding of actin microfilaments over fixed Integrin along osteocyte processes suggests that these abundant connections between the canalicular wall and osteocyte processes can amplify the axial strains applied to osteocytes by two orders of magnitude compared with whole-tissue strain.^22^ As a result, low tissue strain in bone from daily activities can be greatly amplified at the single-cell level and stimulate follow-up actions in osteocytes.

In this review, three main aspects of osteocyte mechanobiology will be discussed in detail. The first aspect concerns methodologies commonly used in the study of osteocyte mechanobiology. Second, how osteocytes sense and transduce mechanical signals into biochemical signals is still debated in the field. Increasing evidence regarding different aspects of osteocytes suggests that osteocytes utilize various molecular mechanosensors to achieve force adaptation. In this review, the seven major mechanosensors reported so far, osteocyte cytoskeletal components, osteocyte dendrites, FAs, gap junctions (GJs), the primary cilium, ion channels, and the ECM, will be introduced and discussed in great detail (Fig. 1c). Third, several mechanotransduction-associated signaling pathways recently reported in osteocytes will be presented and discussed at the end.

## Methodology in osteocyte mechanobiology

To mimic different mechanical stimuli that osteocytes experience under different conditions, various methods to examine osteocyte responses in terms of molecular changes, subcellular deformation and overall bone homeostasis have been developed. Considering the complexity of the physical environment; the diverse stimuli that osteocytes experience; and the different components of certain factors, such as magnitude, frequency, and strain rate, simplified methods have been used both in vitro and in vivo to dissect different biophysical environments and separate individual mechanical stimuli applied to osteocytes. Basically, two major approaches are commonly used in this field.^24^ One is direct mechanical challenge of intact bone tissue in vivo (Tables 1 and 2), while the other takes advantage of in vitro culture systems and involves subjecting osteocytes to different mechanical stimuli (Table 3).

**Table 1:** Experimental conditions for in vivo loading models

Animal	Age and gender	Limb	F	Peak force	Cycles	Durations	Brief summary	Ref.
Ulna loading								
Mice	16 weeks, male and female	Ulna	2 Hz	1.0, 1.2, 1.4, 1.6, 1.8 N	60 per day	3 days	Both BV/TV and BFR/BS were lower in Lrp5-KO femora than in their control femora.	^157^
Mice	16 weeks, female	Ulna	2 Hz	1.90–2.5 N	120 per day	3 days, 16 days	Loading induced the strain-dependent increase in bone formation in WT mice, resulting from increases in both mineralizing surface (MS/BS) and mineral apposition rate (MAR).	^30^
Mice	17 weeks, male and female	Ulna	4 Hz	3.0 and 4.3 N	10 min (strain rate of 0.1 sec)	5 days per week for 2 weeks	Loading to peak strains of 2 000 mu epsilon stimulated lamellar periosteal bone formation, but no response endosteally. Loading to peak strains of 3 000 mu epsilon induced a mixed woven/lamellar periosteal response and lamellar endosteal bone formation.	^33^
Mice	17 weeks, n/a	Ulna	4 Hz	2 N	2 400 per day	10 days	After loading, the increase of cortical bone thickness was detected in the ulna of both Frzb-KO and control mice.	^207^
Mice	18 weeks, male	Ulna	2 Hz	2.7 N	360 per day	2 days	Bone-formation rate are in parallel with strain loading intensity; reduced sclerostin expression in osteocytes, particularly in high strain region/midshaft; reduced Sost, Dkk1 mRNA in loading ulnar.	^25^
Rats	9 weeks, male	Ulna	2 Hz	n/a	1 200 per day	4–8 days and 11–15 days	Approximately 90% of the strain produced by axial loading occurs as a result of medial-to-lateral bending, with the remainder due to axial compression.	^208^
Rats	9 weeks, male	Ulna	2 Hz	n/a	1 200 per day	1–5 and 8–12 days	Least squares regression analysis demonstrated a highly significant linear relationship between the magnitude of the applied load and the degree to which longitudinal growth was reduced.	^209^
Rats	27 weeks, female	Ulna	2 Hz	17 N	360 per day	3 days per week, for 16 weeks	The loaded ulnas exhibited 5.4% and 8.6% greater BMD than the control ulnas in the 360 × 1 and 90 × 4 groups, respectively. BMC was increased by 6.9% and 11.7% in the loaded ulnas of the 360 × 1 and 90 × 4 groups, respectively.	^29^
Rats	17 weeks, female	Ulna	4 Hz	3.0 and 4.3 N	10 min	5 days per week for 2 weeks	The loading-induced periosteal response increased cortical bone area by 21% ± 4% compared with 0.03% ± 0.02% in controls.	^33^
Rats	20 weeks, female	Ulna	4 Hz	20 N	One single stopping point	n/a	Fatigue loading and microdamage formation: Increased TURNL+/Bax+ cells around microdamage, Increased Bcl-2+ cells 1–2 mm away from microdamage.	^175^
Rats	24 weeks, female	Ulna	2 Hz	17 N	360 per day	2 days	Sost transcripts and sclerostin protein levels were dramatically reduced by ulnar loading.	^25^
Tibia loading								
Mice	10 weeks, male and female	Tibia	4 Hz	11.5 ± 0.3 and 2.3 ± 0.3 N	1 200 per day	5 days per week, for 2 weeks	Cancellous BV/TV increased 73% in the loaded tibias relative to control tibias. Mean Tb.Th increased (+75%) while Tb.Sp decreased (−19%). Increased tBMD (+18%) contributed to greater bone mass in the loaded tibias following 2 weeks of compression.	^35^
Mice	10 weeks, male	Tibia	n/a	3 N	1 200 per day	5 days per week, for 2 or 6 weeks	The mineral content in both cortical and cancellous bones was enhanced after 6 weeks of loading. Greater responses were found in the cortico-cancellous proximal metaphysis (14%) than the cortical midshaft (2%); bone volume fraction and average trabecular thickness of cancellous bone in the proximal tibia increased after 6 weeks by 15% and 12%, respectively.	^34^
Mice	12, 14 weeks, male	Tibia	0.1 Hz	12 N	40 per day	3 days per week, for 2 weeks	At the periosteum, loading increased the BFR 15.5-fold and the mineralization perimeter (MPm/BPm) 8.5-fold in control mice.	^210^
Mice	16 weeks, n/a	Tibia	2 Hz	9.3 ± 0.9 N	60 per day	5 days per week, for 2 weeks	Parameters of new bone formation (i.e., MAR, BFR, and MS) were significantly higher in WT than in cKO tibias after the 2-week loading regimen, with Het mice falling somewhat in between the other two groups.	^98^
Mice	16 weeks, n/a	Tibia	2 Hz	9.3 ± 0.9 N	36 per day	6 days per week, for 2 weeks	The bone response to external loading is greater in LBD mice than in HBD mice. The high bone density of C3H/HeJ (HBD) mice is related to breed-specific factors other than the response to loading.	^211^
Mice	19 weeks, female	Tibia	10 Hz	13.5 N	40 per day	n/a	Different region of loaded tibiae responded to loading with different loading-related increases of new bone formation. Among all regions, region-III reached a 75-fold increase. Moreover, the magnitude of loading-related decrease in the percentage of sclerostin-positive osteocytes mirrored the amount of loading-related osteogenesis.	^212^
Mice	13–19 weeks female	Tibia and ulnae	10 Hz	12.0 N for tibia and 2.5 N for ulna	40 per day	10-s intervals between each cycle, for 2 weeks	In trabecular bone of the proximal tibiae, 2 weeks of mechanical loading sufficient by itself to stimulate an osteogenic response, was associated with a 18.6% increase in percent bone volume in the primary spongiosa, a 31.9% increase in percent bone volume in the secondary spongiosa, and a 13.1% increase in trabecular number and a 15.8% increase in trabecular thickness.	^213^
Mice	8, 12, 20 weeks, female	Tibia	2 Hz	2–13 N	40 per day	10-s intervals between each cycle, for 2 weeks	For 12- and 20-week-old mice, loading induced significant decreases in BV/TV. In contrast, tibiae of younger 8-week-old mice show significant increases in BV/TV, achieved predominantly via increases in trabecular number.	^27^
Mice	10, 26 weeks, male	Tibia	4 Hz	4.5/9.0 N	1 200 per day	1, 2, and 6 weeks	In both adult and young mice, loading at 9 N decreased epiphyseal bone mass with a greater decrease observed in the adult mice compared with the young mice. Bone mass increased by 20% with loading in young mice.	^31^
Mice	26 weeks, female	Tibia	4 Hz	11.3 ± 0.5, 5.9 ± 0.5, and 1.5 ± 0.6 N	1 200 per day	5 days per week, for 2 weeks	After loading, cancellous bone mass increased 54% through trabecular thickening, and cortical area increased 41% through medullary contraction and periosteal expansion. Adult mice were able to respond to an anabolic stimulus and recover bone mass to levels seen in growing mice; however, the adaptive response was reduced relative to that in 10-week-old female mice for the same applied load.	^32^
Rats	36 weeks, female	Tibia	2 Hz	27, 33, 40, 52, and 64 N	36 per day	12 days	Bending strains above a loading threshold of 40 N or about 1 050 mu strain increased both bone-forming surface and the mineral apposition rate and subsequently increased the bone-formation rate as much as six folds. No evidence of increased bone formation was seen for applied strains below 1 050 mu strain.	^170^

*F* frequency for loading, *Ref*. references, *n/a* not available

**Table 2:** Experimental conditions for in vivo hindlimb unloading models

Animal	Age and gender	Tail-suspension durations	Brief summary	Ref.
Mice	6 weeks, male	3 days or 7 days	Increased Sost mRNA in unloading tibia after 3 days treatment; no significant sclerostin-positive osteocytes detected in unloading groups.	^25^
Mice	12 weeks, female	21 days	Hindlimb bone mineral density decreased 9.2% ± 1.0% in HLU of control group.	^28^
Mice	17–21 weeks, female	14 days	Myonuclear number was not altered during either the suspension or the reloading period in soleus muscle fibers from vehicle-treated or satellite cell-depleted animals.	^36^
Mice	5 months, n/a	10 days	During the unloading period, soleus muscle fiber cross section decreased by 38%.	^37^
Mice	14 weeks, male	28 days	28 days of HLU-induced serious damages in microstructure and mechanical property of the tibia in WT mice.	^123^
Mice	3 months, female	21 days	HLU-induced significant bone loss, as demonstrated by significant decreases in BV/TV, Tb.N, and Ct.Th and an increase in Tb.Sp.	^169^
Mice	4 months, n/a	3 or 14 days	The soleus muscle/body weight ratio decreased by 41% in WT-HLU 14 days, whereas cross‐sectional area fell by 29% in WT‐HLU 14 days.	^38^
Mice	8 weeks, male	21 days	The mRNA expression of Lcn2 significantly increased in the bones of the suspended hindlimbs with respect to those of the hindlimbs of mice maintained under normal conditions.	^214^
Rats	6/8 months, female	28 days	After a 28-day protocol, disuse group reduced BFR (−92%), a suppression only slightly curbed when disuse was interrupted by 10 min of weight bearing (−61%). In contrast, disuse interrupted by 10 min per day of low-level mechanical intervention normalized BFR to values seen in age-matched controls.	^26^
Rats	9/29 months, male	14 days	Decreases in body weight were observed between the adult (12.2% loss) and old (14.6% loss) rats through 14 days of HLU. Adult rats lost a greater percentage of their hindlimb muscle mass after 2 weeks of HUL compared with the old rats.	^215^

*F* frequency for loading, *Ref*. references, *n/a* not available, *BV/TV* bone volume fraction, *Tb.N* trabecular number, *Ct.Th* cortical thickness, *Tb.Sp* trabecular separation, *BFR* bone-formation rate

**Table 3:** Experimental conditions for in vitro mechanical loading models

Cell type	Shear stress/Pa	Flow type	Flow duration	mRNA changes	Other responses	Ref.
Primary osteocytes						
Chicken primary osteocytes	0.5	p	1 h	*PGE*_*2*_↑	All three cell populations rapidly (osteocytes: within 5 min, osteoblast and osteocyte containing population, periosteal fibroblasts: within 10 min) increased their release of prostaglandins E2 and I2 in response to PFF, but the response by osteocytes was 2–4 times higher than that by osteoblast and osteocyte containing population or periosteal fibroblasts.	^39^
Chicken primary osteocytes	0.7	p	10 min	*PGE*_*2*_↑	PFF raises intracellular Ca^2+^ by an enhanced entry through mechanosensitive ion channels in combination with Ca^2+^ and inositol trisphosphate-induced Ca^2+^ release from intracellular stores.	^216^
Mouse primary calvarial bone cell	0.70 ± 0.03	p	1 h	*PGHS-2*↑	Northern blot analysis detected after 1 h of PFF treatment increased PGHS-2 mRNA expression about twofold; more PGE_2_ was released under PFF condition.	^40^
Human primary trabecular bone cell	0.7	p	1 h	*PGE*_*2*_↑	Cultured cells responded to mechanical stress with enhanced release of prostaglandin E2 (PGE_2_) and I2 (PGI_2_) by western blot.	^105^
Human primary bone cells	0.7	p	1 h	*Cox-2*↑*PGE*_*2*_↑	One-hour PFF treatment stimulated the release of PGE_2_ by 3.5 folds and PGI_2_ by 2.2-fold. PFF also increased the expression of *Cox-2* mRNA by 2.9 folds, but did not change *Cox-1* mRNA by QPCR.	^217^
Human primary bone biopsies cells	0.7	p	1 h	NO↑*PGE*_*2*_↑	The PFF-mediated upregulation of PGE_2_ release during 24 h of postincubation after 1 h of PFF was significantly reduced in osteoporotic patients compared with six age-matched controls as well as with the whole nonosteoporotic group.	^41^
Osteocyte-like cell lines						
Ocy454	0.5–2.0	Un-L	2 h or 3 days	*Rankl*↓*Sost*↑	Ocy454 cells recapitulated the in vivo response to mechanical unloading with increased expression of *Sost* (3.4 ± 1.9-fold), Sclerostin (4.7 ± 0.1-fold), and the receptor activator of *Rankl/Opg* (2.5 ± 0.7-fold) ratio.	^43^
MLO-Y4	0.5–5.0	o	1–4 h	*Rankl*↓*Opg*↓*Cox-2*↑	OFF stimulation simultaneously upregulated the *Cox-2* mRNA expression and downregulated the *Rankl/Opg* mRNA levels.	^42^
MLO-Y4	0.7	p	1 h	*Rankl/Opg*↓*Opg*↑ *MEPE*↑	PFF upregulated MEPE gene expression by 2.5-fold, but not PHEX expression. PFF decreased the *Rankl/Opg* ratio at 1-h PFF treatment.	^218^
MLO-Y4	16.0	s	0.5–2 h	*Opg*↑	MLO-Y4 cells plated at lower densities release more PGE_2_ than cells plated at higher densities. Cell surface biotinylation analysis showed that surface expression of Cx43 was increased by shear stress.	^105^
MLO-Y4	16.0	s	0.5–2 h	*Cx43*↑	SFF has stimulatory effects on MLO-Y4 cells with early effects on cellular morphology, opening of gap junctions, and redistribution of Cx43 protein and delayed effects on Cx43 protein expression.	^102^

*P* pulsating, *s* steady, *o* oscillating, *Un-L* unloading, *PFF* pulsating fluid flow, *SFF* steady laminar fluid flow, *OFF* oscillating fluid flow, *PGE* prostaglandins, *PGHS* prostaglandin G/H synthase, *COX* cyclooxygenase, *RANKL* receptor activator of nuclear factor kappa-Β ligand, *OPG* osteoprotegerin, *MEPE* matrix extracellular phosphoglycoprotein, *PHEX* phosphate-regulating neutral endopeptidase, *NO* nitric oxide, *CX43* connexin-43, *Ref*. references

**Fig. 2:**
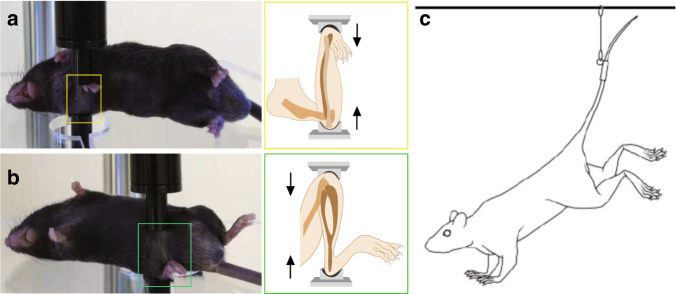
In vivo models commonly used in osteocyte mechanobiology. Examples of active loading models with the right ulna (**a**) and right tibia (**b**) in mice. Loading model mice were under avertin-induced anesthesia, and the right sides of the ulna and tibia were subjected to cyclic mechanical compression with a computationally controlled machine. The contralateral left limbs served as controls. Illustrations of ulna and tibia loading are highlighted in yellow and green boxes, respectively. **c** Illustration of the HLU model in mice.^25^ Experimental mice were outfitted with a tail harness, and their hindlimbs were suspended within customized cages. [panel **c** from Robling et al.^25^, reprinted with permission]

Direct in vivo mechanical stimulation is mainly applied to small experimental animals, such as mice and rats, in which gene manipulation and the recapitulation of mammalian bone features are easy. Both active loading models and unloading models are applied in these animals. To develop models of active loading, experimental animals are maintained under isoflurane- or avertin-induced anesthesia, and one side of either the tibia or ulna is subjected to cyclic mechanical compression under a computationally controlled machine (Fig. 2a, b). The contralateral unloaded limb serves as a control. This cyclic compression somehow mimics the process of force generation from physical activities, such as grabbing, walking or running, and contributes to forces applied to bone osteocytes. Hindlimb unloading (HLU), which focuses on disuse conditions and mimics bone loss induced by trips to space and decreased activity, is also popular in studies. In these experiments, animals are outfitted with a tail harness, and their hindlimbs are suspended within customized cages (Fig. 2c). The mice use their forelimbs to contact the cage floor to obtain food and water, but their hindlimbs remain suspended in the air and lose ground reaction forces. These in vivo animal model systems help us to understand the direct relationship between force application and bone adaptation.

In vivo studies provide strong evidence for “Wolff’s law,” which demonstrates significant bone formation under force application (Table 1)^25–27^ and bone loss under unloading conditions (Table 2).^25,26,28^ These in vivo studies further suggest that bone adaptation is dependent on the animal species, animal age, loading site, and magnitude and duration of the force applied (Table 1). For example, considering differences in the mechanical properties of limbs between different species, the average peak force used for ulna loading in rats is ~17 N,^25,29^ while that used in mice is only 2–4 N.^25,30^ Moreover, considering the different physical loading forces applied on different limbs within the same species, the average external force applied for mouse tibia loading is ~9–11 N,^25,29^ while that applied for mouse ulna loading is only 2–4 N.^25,30^ Moreover, force loading must be kept in a particular physical range for different experimental subjects, and extreme force loading could promote cartilage damage and reduce cancellous bone mass in both young and old mice.^31^ Furthermore, loading effects vary with age. For instance, compared with 10-week-old (young) female mice, 26-week-old female mice displayed less new bone formation when the same load was applied to the tibia.^32^ In addition, forces with different magnitudes and durations generate various outcomes. When a peak force of 3 N (a peak strain of 2 000 mu epsilon) was applied to the mouse ulna, only lamellar periosteal bone formation was stimulated, but no endosteal response was observed. Similarly, when with a peak force of 4.3 N (a peak strain of 3 000 mu epsilon) was applied to the mouse ulna, a mixed woven/lamellar periosteal response and lamellar endosteal bone formation were induced.^33^ Moreover, animals to which tibia loading was applied for 6 weeks showed a more significant enhancement in mineral content in both cortical and cancellous bones compared with that after continuous force application for 2 weeks.^34^ Interestingly, bone formation from mechanosensory osteocytes seems to be independent of sex. Lynch et al. showed that cancellous bone adaptations to tibia compression in growing male and female mice were comparable.^35^

While results from in vivo loading and unloading experiments provide strong evidence of bone phenotypes under different force conditions, contributions from other cell types during mechanical loading or unloading and the effects from surrounding tissues during experiments cannot be ruled out.^36–38^ Therefore, in vitro studies focused on osteocytes, a single cell type, can rule out the influence of other cell types and assess osteocyte mechanobiology in greater detail. In general, two types of osteocytes are commonly used in in vitro loading studies (Table 3). One type is primary osteocytes, which are obtained from bone tissues, such as mouse calvarial bone, the chicken skeleton, or even human trabecular bone, through sequential enzymatic digestion.^39–41^ The other cell type is osteocyte-like cell lines transformed from primary osteocytes that exhibit immortalization, such as MLO-Y4 cells^42^ and Ocy454 cells.^43^ These two cell lines are widely used osteocyte-like cells that express specific osteocyte markers and whose morphology resembles that of primary osteocytes. Several physiologically relevant signaling molecules, including intracellular Ca^2+^, IP3, cAMP, prostaglandin E_2_ (PGE_2_), and nitric oxide (NO), are known to be secreted when osteocytes are supplied with fluid flow in vitro (Table 3). Moreover, cell culture studies can recapture in vivo experimental results, such as increased sclerostin expression under unloading conditions.^43^ These results demonstrate that in vitro methodology is highly valuable for investigating osteocyte responses to loading and unloading stimulation.

Currently, more advanced technologies, including those performed in in vivo such as three-dimensional (3D) fluorescence imaging,^44^ ex vivo live calcium recording,^45^ and in vitro inventions, such as bone chip organ culture,^46,47^ provide more advanced choices to study osteocyte mechanobiology. Many essential aspects of osteocytes in force adaptation are revealed by in vitro techniques and further demonstrated in in vivo models. We will discuss these methodologies in more detail in the following sections regarding osteocyte mechanosensors and signaling pathways.

## Osteocyte mechanosensors

How osteocytes sense external mechanical environments, convert mechanical signals into internal biochemical signals, and eventually transduce these signals into different biological functions remains intriguing. The special cellular components or proteins that carry out this signal transduction are called mechanosensors. Over the past two decades, great efforts have focused on finding these mechanosensors. The results of these studies suggest that osteocytes utilize various mechanosensors to respond to physical stimulation. Here, we will introduce and discuss the seven mechanosensors reported so far in detail.

### Cytoskeletons: actin filaments, microtubules, and intermediate filaments

**Fig. 3:**
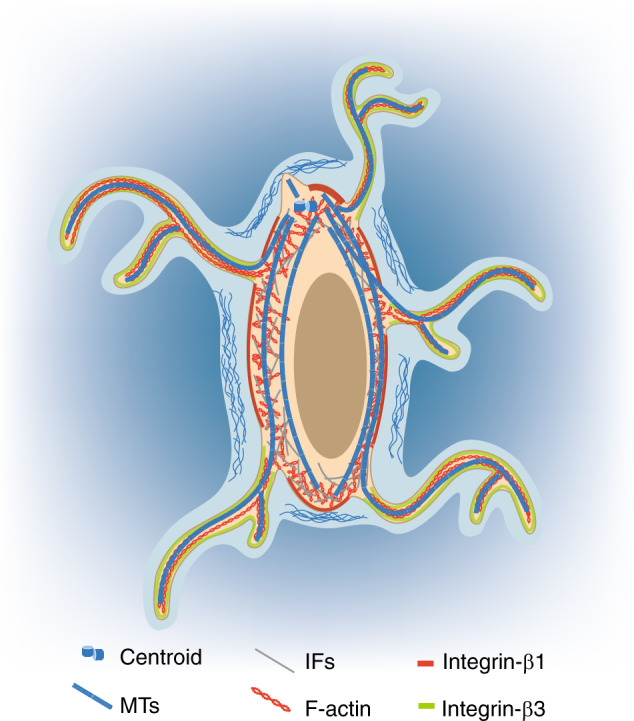
Illustration of cytoskeleton and Integrin subunits in a single osteocyte. Three types of cytoskeletal components are observed in osteocytes^52^: IFs are mainly reported in the cell body, and F-actin and MTs are reported in both the cell body and dendrites. Compared with total MTs, detyrosinated MTs seem to be more localized to osteocyte processes and the primary cilium.^53^ The focal adhesion proteins Integrins show distinct distributions in osteocytes^20^: Integrin β1 is mainly localized to the plasma membrane of the cell body, whereas Integrin β3 is primarily localized to the surface of dendrites

All cells are considered viscoelastic materials that can change shape under mechanical load.^48^ The origin of the mechanical properties of cells and their responses to extracellular mechanical stimuli are mainly dependent on the cytoskeleton, especially the mechanical properties of the cytoskeleton.^49^ For osteocytes, three types of cytoskeletal filaments define the cell: actin filaments (F-actin), microtubules (MTs), and intermediate filaments (IFs). The mechanical properties of these cytoskeletal components, together with the filament length, crosslinking geometry, and host binding proteins to the side or at the end of cytoskeletal polymers, determine the mechanical properties of cytoskeletal networks and cells^50^ (Fig. 3). A computational model based on three cytoskeletal components and cell-ECM connections in osteocytes demonstrated a possible mechanism of mechanotransduction from extracellular mechanical stimuli to nuclear responses.^51^

Even though three types of cytoskeletal filaments contribute to the mechanical properties of the cell, their functions regarding osteocyte mechanical responses differ. In primary osteocytes derived from chicken embryos, immunofluorescence (IF) staining with antitubulin, antivimentin, and antiactin antibodies suggested that the distribution patterns of these cytoskeletal components differ.^52^ All three filaments are detected in the osteocyte cell body, but only F-actin is located in osteocyte processes. Two actin-bundling proteins, α-actinin and Fimbrin, colocalize in osteocyte processes. Depolymerization of F-actin using pharmaceutical drug treatment (latrunculin B and cytochalasin D) significantly changed the osteocyte morphology, including the retraction of processes and a decrease in the overall size of the cell body until it was a cytoplasmic rim around the nucleus, leaving the cell membrane with folds and wrinkles. These results reveal the great importance of F-actin in the maintenance of osteocyte morphology, membrane tension and mechanosensory capability.

In addition to F-actin, MTs participate in osteocyte mechanosensation. A more recent study performed by Lyons et al. showed that MTs are involved in calcium flux and sclerostin expression in osteocytes.^53^ Unlike total MTs, detyrosinated MTs were localized to osteocyte processes and the primary cilium in both primary murine osteocytes and the Ocy454 osteocyte cell line. Intact MTs were found to be required for the response of Ocy454 cells to fluid shear stress (FSS), including calcium flux and the regulation of sclerostin expression. Moreover, the MT network is also essential for flow-induced opening of the Ca^2+^ channel TRPV4 and Ca^2+^ influx. During these processes, NADPH oxidase 2 and reactive oxygen species participated in MT-dependent CaMKII kinase activation and sclerostin suppression under FSS conditions. These data suggest the role of MTs in the regulation of calcium channel opening and gene expression during osteocyte mechanotransduction.

Fewer studies have been performed on IFs in osteocytes than on F-actin and MTs. Moorer et al. generated transgenic mice in which Synemin, a type IV IF protein, was globally deleted.^54^ Synemin-null animals displayed normal development with body weights, body lengths, and tibial lengths comparable with those of control animals. At the age of 14 weeks, male Synemin knockout (KO) mice displayed a dramatic osteopenic phenotype in the trabecular bone and a subtle reduction in the cortical area in the femur bone. Further ELISA and in vitro experiments suggested a significant reduction in osteoblastic bone-formation activity and a reduction in osteoblast number in Synemin-KO mice. Interestingly, the expression of osteoblast-related genes, such as those encoding Runx2 and Osteocalcin (Oc), was increased in primary osteoblasts isolated from Synemin-KO mice. These results suggest the potential role of IFs in the regulation of osteogenesis. Further experiments are required to show the relationship between IFs and osteocyte mechanotransduction.

In short, as the major building blocks in osteocytes, together, these three types of cytoskeletal filaments maintain normal osteocyte morphology and regulate osteocyte responses to mechanical stimulation. Some special structures generated from the osteocyte cytoskeleton, such as the primary cilium generated from MTs and FAs associated with F-actin, have been widely acknowledged as the major mechanosensors in osteocytes. More studies on IFs in osteocytes are required. Even though these types of filaments have different functions in osteocyte biology, it would be interesting to examine the interactions and communication between different types of cytoskeletal filaments in osteocyte mechanobiology.

### Dendrites vs the cell body

**Fig. 4:**
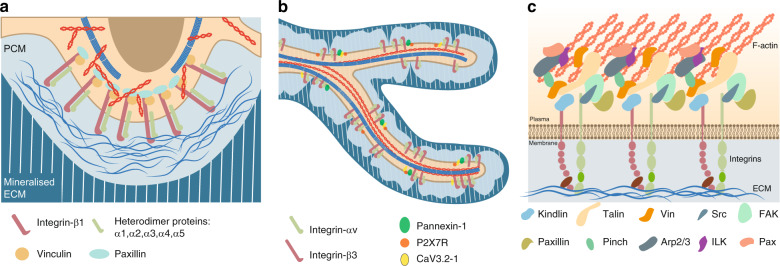
Focal adhesions on the osteocyte cell body and dendrites. **a** Heterodimers of Integrin β3 with Integrins α1/2/3/4/5 are localized to the osteocyte cell body surface.^23^ The heads of these heterodimers contact the pericellular matrix, and their tails are linked to the F-actin cytoskeleton. Moreover, the classical focal adhesion components Vinculin and Paxillin were reported to colocalize with Integrin β1-mediated focal adhesions.^83^**b** Heterodimers of Integrin β3 with Integrin αv are localized to osteocyte dendritic surfaces.^23^ The purinergic channel pannexin 1, the ATP-gated purinergic receptor P2X7R and the low-voltage transiently opened T-type calcium channel CaV3.2-1 reside in close proximity to Integrin β3 attachment foci.^83^**c** Illustration of major focal adhesion components at cell-ECM interphases.^165,166^

The feature of osteocytes that most distinguishes them from other bone cells is the tremendous dendritic processes that emerge from the osteocyte cell body (Fig. 1). The cell body and dendrites are similar to other specialized cellular compartments and have been assigned different roles in osteocyte mechanobiology (Fig. 4). Among attempts to locate and specify the mechanosensors in osteocytes, debates regarding where osteocytes sense and respond to mechanical stimulation have been ongoing for years.

Compared with the cell body, the dendritic processes of osteocytes are more inclined to be related to mechanotransduction (Fig. 4b). The formation and elongation of dendrites are highly associated with mechanical stimulation.^55^ These processes are regulated by a glycoprotein named E11 or Gp38 that is selectively expressed in osteocytes.^55^ E11/Gp38, also called podoplanin or T1alpha molecule, is highly expressed in the dendritic processes of early osteocytes that have begun to be embedded in osteoid but is not expressed in fully differentiated osteocytes in the mineral matrix. ECM mineralization is proposed to be tightly linked to osteocyte differentiation through the E11/Gp38 protein. On the one hand, the physical properties of the ECM can influence osteocyte differentiation by regulation of E11/Gp38 expression.^56^ On the other hand, mechanical strain both in vitro and in vivo increases E11/Gp38 expression in osteocytes.^55^ The fluid flow shear stress-induced elongation of dendritic processes in MLO-Y4 cells requires E11/Gp38. However, increased E11/Gp38 expression under mechanical stimuli is not beneficial under certain conditions. Increased E11/Gp38 expression in osteocytes was observed in human and canine osteoarthritic (OA) subchondral bone,^57^ which resulted in subchondral bone thickening and served as an early detectable marker in osteoarthritis (OA) joints. Moreover, conditional deletion of E11/Gp38 in mature osteoblasts and osteocytes (*OC-Cre; E11*^*fl/fl*^) prevented mechanical load-induced articular cartilage lesions.^57^ Together, these data suggest that E11/Gp38 participates in osteocyte differentiation and mineralization and that E11/Gp38-associated dendritic process growth is involved in osteocyte mechanotransduction.

In addition to these studies focused on osteocyte dendrite generation, several research groups have utilized different methodologies to directly examine differences in the responses of the cell body and dendritic processes under mechanical stimuli (Fig. 4a). Burra et al. generated a transwell filter system on which MLO-Y4 cells were cultured.^58^ Due to its specific 1-μm pore size, the filter system in this system could separate the MLO-Y4 cell body and dendritic processes. When fluid droplets were applied to the cell body side or the dendritic side, different parts of the cultured cells experienced mechanical stimulation. Interestingly, when mechanical loading was applied to either the dendrites or the cell body, opening of the hemichannels on the cell body was induced, but the hemichannels on the dendritic side showed no significant activity under either treatment. Moreover, when the glycocalyx on the dendritic side was disrupted by hyaluronidase, hemichannel opening on the cell body was completely blocked. IF staining showed reduced Integrin α5 intensity on osteocyte dendrites after hyaluronidase treatment. These observations suggest that the hemichannels on the cell body side are the channels responsive to mechanical stimulation and that mechanical stress applied to cell dendritic processes requires strong Integrin-based attachment to the extracellular glycocalyx. This Integrin–glycocalyx interaction along osteocyte processes works as a mechanosensor that transmits mechanical signals from cell dendrites to the cell body and leads to the opening of hemichannels (please see below).

Another research team utilized a local fluid stimulation approach to investigate the relationship between cell dendrites and the cell body in response to mechanical force in osteocytes.^59,60^ This approach uses what is called a Stokesian fluid stimulus probe, which can generate local fluidic stimulation of 1–5 pN without physical contact with the examined cells. Thi et al. presented direct evidence that mechanical force stimulation of dendrites, but not the cell body, activate directional calcium flow in osteocytes.^60^ Preincubation with a nonpeptide small-molecule ανβ3 Integrin antagonist or an extracellular ATP scavenger (Apyrase, an enzyme that hydrolyzes ATP to AMP) reduced the amplitude and percentage of cellular Ca^2+^ responses. In addition, Wu et al. showed that focally applied pN-level forces initiated rapid and transient intercellular electrical signals in cultured MLO-Y4 cells.^59^ This electrical signal was triggered at Integrin attachment sites along both appositional and distal unopposed cell processes but was not initiated at osteocyte cell bodies. This electrical coupling required the presence of ATP released by the stimulated cells, and its strength was increased with increasing numbers of junctional connections, which have been reported to be regulated by Cx43 (detailed information about Cx43 will be discussed in the following section).

Due to the efforts from these brilliant research groups, it is now well accepted that environmental mechanical stimuli sensed by osteocytes are largely relayed through the dendritic processes of osteocytes rather than osteocyte cell bodies (Fig. 4). Osteocytes utilize dendritic processes to receive mechanical signals, transduce these signals through the F-actin and MT cytoskeleton or electrical or calcium signals, and initiate nuclear responses to regulate the expression of target genes (e.g., *Sost*) or initiate responses from certain subcellular organelles with secondary messenger (e.g., Ca^2+^ and ATP) activation. It would be interesting to compare osteocyte processes and neuron dendrites. In neurons, signal transmission both in a single neuron and within neuronal networks occurs in a highly organized and directional manner through certain physical functions.^61^ It would be worth testing whether osteocytes respond to mechanical stimulation with similar directional signal transduction through their processes.

### Primary cilia

Cilia are located on the surfaces of almost all mammalian cells.^62^ The typical structure of the cilium consists of a central MT-based axoneme that emerges from a centriole-derived, MT-organizing center called the basal body and extends from a specialized plasma membrane into the extracellular space.^62^ Unlike other types of cilia, the primary cilium is a special, solitary organelle that projects from the surface of certain cells. The primary cilium consists of nine doublet MTs but lacks the central pair of MTs needed to generate motile force; the axoneme thus adopts a “9 + 0” pattern.^62,63^ The axoneme is formed and maintained by intraflagellar transport (IFT) complexes that are trafficked through the ciliary axoneme by the molecular motors anterograde kinesin-II and retrograde dynein 2.^63,64^ During the cell cycle, the primary cilium can be reabsorbed before the cell enters mitosis and reformed through acetylation of α-tubulin when the cell enters the quiescent G0/G1 stage.^64^ Since primary cilia exhibit special structural features and directly contact the extracellular environment, it is widely believed that the primary cilium plays pivotal roles in chemosensation and mechanosensation.^65^

In the bone tissue environment, the primary cilium has been found in large groups of cells, such as bone-derived mesenchymal stem cells (MSCs), tenocytes, chondrocytes, and osteocytes.^65^ In human bone-derived MSCs, disruption of the primary cilium with *Polaris* (an IFT-associated protein) siRNA treatment reduced mechanically stimulated *Cyclooxygenase-2* (*Cox-2*) and *Bone morphogenetic protein 2* (*Bmp2)* mRNA expression.^66^ During chondrocyte development, conditional deletion of *Smad1/5* in chondrocytes altered the 3D orientation of the primary cilium without affecting the primary cilium length.^67^ As a result, misorientation of the primary cilium further affected chondrocyte cell positioning during cell division, caused the misalignment of chondrocytes in columns, and eventually resulted in disorganized growth plates in *Smad1/5* conditional KO (cKO) mice.^67^

**Fig. 5:**
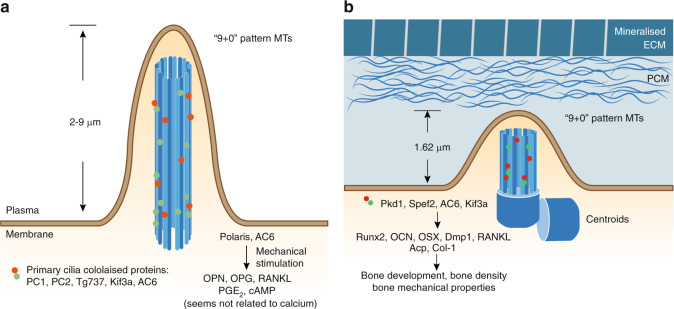
The osteocyte primary cilium in mechanobiology. **a** Illustration of the primary cilia from in vitro cultured osteocyte-like cells. The primary cilium is a unique cell protrusion structure consisting of nine doublet microtubules in the form of a “9 + 0” pattern.^62,63^ In cultured MLOY4 cells, this cilia-like structure was shown to be 2–9 μm in length.^68,69^ Several ciliary proteins, such as PC1, PC2, Tg737, and Kif3a, colocalize in this structure.^68^ Among them, Polaris and AC6 were reported to participate in osteocyte responses to mechanical stimulation.^72^**b** Illustration of the primary cilium in vivo from the embedded osteocytes of bone sections. Unlike the results of in vitro detection, in vivo recordings of the primary cilium showed a morphological change of the cell membrane in which the mother centriole contacts the plasma membrane and a very short axoneme forms a cilium-like protrusion.^70^ With Aα-Tub staining and confocal imaging, primary cilia in osteocytes were measured and found to have an average length of 1.62 μm.^71^ The ciliary proteins Pkd1,^68^ Spef2,^73^ AC6,^76^ and Kif3a^74^ also participate in osteocyte mechanical bone adaptation

In osteocytes, the primary cilium is an important sensor for the responses to mechanical stimulation and coordinates loading-induced bone adaptation^65^ (Fig. 5). In cultured primary osteoblasts, osteocytes and related cell lines, cilia-like structures were detected through α-Tubulin immunostaining under scanning electron microscopy (SEM).^68^ These structures are colocalized with the ciliary proteins PC1/polycystin-1, PC2, Tg737, and Kif3a (Fig. 5a). In cultured confluent preosteoblast-like MC3T3-E1 cells and osteocyte-like MLOY4 cells, these cilia-like structures had lengths ranging from 2 to 4 μm.^68^ In a similar study, primary cilia 4–9 μm in length were reported on the apical surface of ∼61% of MC3T3-E1 cells and ∼62% of MLO-Y4 cells.^69^ This difference in length may result from different culture conditions and passage numbers.

In addition to in vitro culture conditions, direct observation of the osteocyte primary cilium in bone samples has been achieved in vivo. In a study focused on osteocyte centrosomes and cilia in the adult (6–7 months old) rat tibial cortical bone, positive staining for acetylated α-tubulin (Aα-Tub) was observed in 94% of the osteocytes under confocal microscopy.^70^ This positive staining for Aα-Tub, which indicates the primary cilium, primary cilium-related zone, or centroids, was mainly oriented perpendicular to the long axis of the bone. In this study, Uzbekov et al. analyzed TEM recordings of primary cilia from ultrathin (70-nm) bone sections. Rather than a clear and distinct primary cilium structure, Uzbekov et al. observed primary cilia at only the initial stages formation, named “cilium membrane prolongation (CMP)” (Fig. 5b). The presence of this CMP structure indicates morphological changes of the cell membrane where the mother centriole contacts the plasma membrane, and a very short axoneme was associated with a cilium-like protrusion.^70^ Another study of trabecular bone from the cervical vertebrae of young sheep (6–8 months old) found that primary cilia were widespread in bone samples (marrow space, endosteal surface, mineralization matrix).^71^ Unlike the results of a previous study, only 4.04% ± 1.04% of osteocytes examined in this study presented primary cilia, which were located in the lacunae adjacent to the osteocyte nucleus. With Aα-Tub staining and confocal imaging from sheep bone samples, the measured cilia in osteocytes were found to be 1.62 μm in length on average (Fig. 5b), whereas cilia within the marrow could reach up to 7 μm in length. Discrepancies in primary cilium length and frequency between in vitro and in vivo samples could result from different extracellular environments (2D culture vs LCS), cellular status (immortalized cells vs primary cells), and even sample preparation procedures.^70^ Considering these discrepancies and the difficulties of in vivo primary cilium studies, more efforts are required to achieve better in vivo imaging results to assess the primary cilium under physiological conditions.

Primary cilia have been demonstrated to participate in osteocyte mechanotransduction both in vitro and in vivo. Under culture conditions, primary cilia were reflected in the direction of steady flow of 0.03 Pa and recoiled after cessation under live imaging.^69^ Disruption of the primary cilia structure through either chloral hydrate treatment or *Polaris* siRNA in both the MC3T3-E1 and MLO-Y4 cell lines reduced cellular responses to flow, which included reductions in mechanically induced *Opn* mRNA expression, extracellular PGE_2_ levels and the *Opg/Rankl* mRNA ratio.^69^ However, these treatments for cilia dysfunction caused no changes in cellular calcium flux in MLO-Y4 or MC3T3-E1 cells. Furthermore, MLO-Y4 cells rapidly responded to 1 peak shear stress oscillatory flow of 1 Pa (1 Hz) and exhibited intracellular cAMP levels reduced to 50%–60% in the first 2 min, and then increased to a 1.5-fold higher level after 30 min.^72^ These responses were reversed by A*denylyl cyclase 6* (*AC6*) siRNA, similar to the effects of *Polaris* siRNA. AC6 is a GTP-dependent enzyme responsible for catalyzing the conversion of cAMP from ATP. AC6 was found to localize to the primary cilium in MLO-Y4 cells. Moreover, AC6 is a calcium ion-inhibited isoform of adenylyl cyclase. Depletion of stored intracellular calcium through thapsigargin treatment had no effect on the flow-mediated decrease in cAMP in MLO-Y4 cells. However, blockade of extracellular Ca^2+^ entry through membrane-bound channels using GdCl treatment prevented the flow-mediated decrease in cAMP.^72^ Together, these data suggest that primary cilia influence osteocyte cellular responses to external shear stress through regulating the intracellular cAMP level and extracellular calcium entry independent of intracellular calcium signals.

In addition to in vitro studies, several groups have investigated the function of primary cilium proteins in transgenic animals (Table 4). Because the primary cilium is indispensable for early development, global deletion of *Polycystin-1/Pkd*, a transmembrane protein component of the ciliary mechanosensory complex encoded by the *Pkd1* gene, or inactivating missense mutations of the *Pkd1* gene (*Pkd1*^*m1Bei/m1Bei*^) resulted in embryonic lethality in mice.^68^ Further phenotypic examination showed that *Pkd1*^*m1bei/m1bei*^ homozygous mice displayed delayed endochondral and intramembranous bone formation with reduced *Runx2* expression. Heterozygous (*Pkd1*^*+/m1bei*^) mice showed reduced mineral density, a reduced mineral apposition rate, and reduced expression of osteoblast-related genes encoding *Oc*, *Osterix (Osx)*, *Opg*, and *Rankl* and the osteoclast marker tartrate-resistant acid phosphatase. Moreover, another important cilia-related protein, Sperm flagellar protein 2 (Spef2), is also involved in the bone-formation process.^73^ Compared with control mice, *Spef2-KO* mice had significantly shorter tibiae and femora, with reduced skull bone thickness at P15, lower bone density for both the trabecular and cortical bone of the vertebrae and distal femur, and decreased mechanical properties. Further examination showed that osteoblast differentiation was impaired in *Spef2-KO* mice, with decreased expression levels of *Alp, Runx2, Col1*, and *Oc* (Fig. 5b). Together, these data demonstrate the importance of the primary cilium in bone development.

**Table 4:** Animal studies focused on primary cilium in osteocytes

Targeted cells	Genotype	Baseline skeletal phenotype	External stimulation		Ref.
			Mechanical	Chemical	
Global					
*Pkd1*^*−/−*^		Embryonic lethal.	n/a	n/a	^68^
*Pkd1*^*m1Bei/miBei*^		Embryonic lethal; delayed endochondral and intramembranous bone formation through inhibition of *Runx2* expression.	n/a	n/a	^68^
*Pkd1*^*+/m1bei*^		Reduced mineral density, mineral apposition rate, reduced osteoblast marker expression *(Osteocalcin, Osterix, Opg, Rankl)*, osteoclast marker expression *(TRAP)*.	n/a	n/a	^68^
*Spef2*^*−/−*^		Significantly shorter tibiae and femora length, with reduced skull bone thickness at P15, lower bone density for both trabecular and cortical bone (Th10 and L2 vertebrae and distal femur), and lower mechanical properties; osteoblast differentiation is impaired in the Spef2-KO mice with lower *Alp, Runx2, Col1*, and *Osteocalcin* expressions.	n/a	n/a	^73^
*AC6*^*−/−*^		Normal bone morphology.	Impaired responses to mechanical loading; mice lacking *AC6* had 41% lower bone formation compared with control animals; primary bone cells isolated from *AC6-*null mice had an attenuated flow induced increase in *Cox-2* mRNA expression.	Similar bone formation in responses to osteogenic chemical agents (PTH).	^76^
Osteoblasts and osteocytes					
*Cola1(I) 2.3-Cre; Kif3a*^*fl/fl*^		No differences in embryo size, limb patterning, nor growth plate architecture at E16.5–18.5, and even no effect on skeletal morphology, bone density, nor bone quality in adult animals.	Skeletally mature (16 weeks old) *Cola1(I) 2.3-Cre; Kif3a*^*fl/fl*^ mice exhibit less responsiveness to mechanical ulna loading compared with control mice; displayed significant less bone formation in dynamic histomorphometry analysis.	n/a	^74^
*OC-Cre; Kif3a*^*fl/fl*^		Developed osteopenia by 6 weeks of age; reductions in femoral bone mineral density (22%), trabecular bone volume (42%) and cortical thickness (17%); impaired osteoblast function.	n/a	n/a	^75^

*Ref*. references, *n/a* not available

To investigate the specific function of the primary cilium in skeletal cells, transgenic mice expressing tissue-specific Cre recombinase were used. Temiyasathit et al. used Cre recombinase driven by the 2.3-kb *Collagen1α(I)* promoter to delete *Kif3a*, an IFT protein required for ciliogenesis, in osteoblasts and osteocytes.^74^ Surprisingly, *Cola1(I)-Cre; Kif3a*^*fl/fl*^ transgenic mice showed no marked abnormalities in embryo size, limb patterning, or growth plate architecture at E16.5–18.5 or skeletal morphology, bone density, or bone quality as adults. Similar to this study, Qiu et al. conditionally deleted *Kif3a* in osteoblasts by using *Oc-Cre*,^75^ which is thought to be expressed in mature osteoblasts. Compared with control cells, primary osteoblasts derived from *Oc-Cre; Kif3a*^*fl/fl*^ mice exhibited significant reductions in primary cilia number (by 51%) and length (by 27%) in vitro. Moreover, these *Oc-Cre; Kif3a*^*fl/fl*^ mice developed osteopenia by 6 weeks of age, unlike *Oc-Cre; Kif3a*^*fl/+*^ and *Kif3a*^*fl/fl*^ control mice. The osteopenic phenotypes included reductions in femoral bone mineral density (BMD) (22%), trabecular bone volume (42%), and cortical thickness (17%). In addition, the loss of bone mass in *Oc-Cre; Kif3a*^*fl/fl*^ mice was associated with impaired osteoblast function in vivo, as evidenced by a 54% reduction in mineral apposition rate and decreased expression of *Runx2*, *Osx*, *Oc*, and *Dmp1*. Interestingly, these differences in bone density between cKO and control mice decreased as the animals grew older, as reported in both studies.^74,75^ The effects of gene deletion at different time frames in osteoblasts and osteocytes suggest that the functions of *Kif3a* in the primary cilium are required for osteoblast maturation in bone development.

Aside from its roles in bone development, the primary cilium of osteocytes is also tightly associated with mechanotransduction. Even though *Cola1(I)-Cre; Kif3a*^*fl/fl*^ mice, as discussed above, showed normal skeletal development, skeletally mature (16-week-old) *Cola1(I)-Cre; Kif3a*^*fl/fl*^ mice exhibited less responsiveness to mechanical ulna loading than control mice.^74^ These animals with primary cilium dysfunctions displayed significantly less bone formation by dynamic histomorphometry analysis. Similarly, mice with global *AC6* KO exhibited a normal bone morphology and similar bone formation in response to an osteogenic agent (PTH) but impaired responses to mechanical loading.^76^ After ulnar loading over 3 consecutive days, mice lacking *AC6* exhibited 41% less bone formation than control animals. Moreover, primary bone cells isolated from AC6-null mice showed an attenuated flow-induced increase in *Cox-2* mRNA expression. Together, these in vivo data suggest that an intact primary cilium in osteocytes is required for proper responses to mechanical stimuli.

Over the past decade, more than a dozen disorders in the human population have been reported to be associated with defective ciliary machinery. These primary cilium-related diseases are named ciliopathies.^64^ Ciliopathies affect nearly every major organ and tissue, including the kidney, brain, limb, retina, liver, and bone.^64^ Among ciliopathies, skeletal ciliopathies, such as ATD (Jeune syndrome) and Ellis–van Creveld syndrome, are caused by mutations in MT-associated motors, basal body proteins or transport proteins in primary cilia.^64^ Recently, a skeletal dysfunction named idiopathic scoliosis (IS), a complex pediatric disease of unknown cause that is characterized by abnormal spinal curvature, was reported to be related to abnormalities in the primary cilium. Primary osteoblasts isolated from IS patients showed significantly elongated primary cilia.^77^ Among cultured primary osteoblasts from both IS patients and healthy donors, the average length of the primary cilium was ~2.6–2.8 μm in IS patients compared with 1.9–2.2 μm in the ilia of healthy donors, whereas the percentages of ciliated cells in control and patient samples were similar. Moreover, when these primary osteoblasts were subjected to physiologically relevant shear stress (1 Hz, 1 Pa), compared with cells from healthy donors, cells from IS patients showed reduced expression of *Integrin β1* and *Bmp2* and decreased production of Cox-2 induced by mechanical stimulation.

Together, observation of the cilium structure in osteocytes from transgenic mice with bone defects and the severe phenotype of bone-related ciliopathies indicate the significance of the primary cilium in development and mechanical-related bone homeostasis. However, more direct and convincing evidence for morphological changes of the osteocyte primary cilium under both physiological and pathological conditions is urgently needed.

### FAs: integrins

Cells sense neighboring microenvironments and nanoenvironments through the FA complex, an Integrin-based adhesion complex.^78^ As the central proteins in the FA complex, Integrins are transmembrane receptors whose extracellular domain connects with the ECM and cytoplasmic domain is linked to FA-associated proteins that are further linked to the cytoskeleton.^79^ Integrin-based FA protein complexes help cells explore and respond to different environmental cues, including the chemical and physical properties of the surrounding matrix and mechanical forces applied directly or indirectly to cells. The complexity and modular nature of different adhesion proteins allow cells to respond differently based on extracellular environment changes.

Integrins are heterodimers formed by α and β subunits: the α subunit is responsible for extracellular ligand specificity, while the β subunit contributes to internal signaling pathways.^79^ A total of 24 αβ heterodimeric Integrin family members have been reported so far, and these Integrins are differentially expressed in different tissues based on the various ECM proteins in the tissue environment.^79^ In skeletal tissue, Integrins are ubiquitously expressed in multiple bone cells, including bone marrow stem cells, osteoblasts, osteocytes, and osteoclasts.^23,80–82^

Two major β Integrin subunits, β1 and β3 Integrin, are found in osteocytes.^23^ Integrin β1 associates with the α1, α2, α3, α4, and α5 Integrin subunits, and Integrin β3 is mainly associated with αv Integrin in osteocytes (Fig. 4). Direct observation from immunohistochemistry (IHC) of bone sections showed that Integrin β1 is mainly found on the osteocyte cell body, whereas Integrin β3 is primarily observed on cell processes.^20^ Even though Integrin β1, which located on the cell body, is more abundant in osteocytes, Integrin β3, which is associated with processes, exhibits more special features. TEM imaging of bone sections showed that Integrin β3 forms distinct puncta along osteocyte processes localized to specialized membrane protrusions.^20^ These protrusions along osteocyte processes are in direct contact with the walls of canaliculi, where the canaliculi have projections that contact ECM collagen and glycocalyx. Unlike traditional FA complexes, these β3 Integrins form atypical FA complexes at these contact points. In a study combining IHC with structural illumination by super-resolution microscopy, Cabahug-Zuckerman et al. examined the spatial correlation between Integrin β3 and other proteins in authentic osteocytes in situ.^83^ Large FA proteins, such as vinculin and paxillin, were detected around the cell body but not on the processes. Instead, a specialized mechanotransduction complex was observed on the osteocyte processes. This complex included the purinergic channel pannexin 1, the ATP-gated purinergic receptor P2X7R and the low-voltage transiently opened T-type calcium channel CaV3.2–1, all of which reside in close proximity to Integrin β3 attachment foci. In summary, Integrin β1 forms a traditional FA complex with Vinculin and Paxillin at the osteocyte cell body (Fig. 4a), while Integrin β3 is distributed with special channel proteins along osteocyte processes (Fig. 4b).

The distinct distributions of Integrins β1 and β3 on osteocytes suggests the different contributions of Integrin β subunits to osteocyte biology. Particular focus has been given to the individual functions of Integrins β1 and β3 in bone development and force adaptation.^23,82,83^

Studies have shown that both Integrins β1 and β3 are essential for the mechanotransduction of cultured osteocytes. When MLO-Y4 cells were stably transfected with vector expressing a dominant-negative isoform of the β1 subunit (β1DN) containing only the transmembrane domain and cytoplasmic tail of Integrin β1, the cells had reduced vinculin localization to FA complexes.^84^ Moreover, in response to oscillatory fluid flow, cells expressing β1DN showed significant reductions in fluid flow-induced *Cox-2* gene expression and PGE_2_ release and lost the capability to show a fluid flow-induced decrease in the *Rankl/Opg* ratio. Intriguingly, cells expressing β1DN showed no alteration in mechanically induced intracellular calcium mobilization. Controversially, another study showed that preincubation with a nonpeptide small-molecule Integrins ανβ3 antagonist (IntegriSense 750) or extracellular ATP scavenger (Apyrase, an enzyme that hydrolyzes ATP to AMP) reduced both the amplitude and percentage of Ca^2+^ responses.^60^ Moreover, following the blockade of Integrin β3 or Integrin ανβ3 activity with the corresponding antagonist at 30 min before the application of oscillatory fluid flow, MLO-Y4 cells displayed reduced cell spreading with process retraction, reduced *Cox-2* expression, and low PGE_2_ release with normal expression of *Rankl* and *Opg*.^85^ These results suggest that Integrins β1 and β3 participate in osteocyte FA complex formation and mechanosensation, which may be independent of calcium flux.

In vivo, Integrin β1 has been shown to play inevitable roles in embryogenesis and bone development (Table 5). Global inactivation of *Integrin β1* in mice resulted in embryonic lethality due to dysfunctions of the inner cell mass and collapsed blastocoeles.^86^ Moreover, Integrin β1 was also found to be required for MSC differentiation. Mice with conditional deletion of *Integrin β1* in mesenchymal condensation cells using *Twist2-Cre* transgenic mice did not survive beyond birth.^87^ These *Twist2-Cre; Integrin β1*^*fl/fl*^ mice had impaired skeletal development, especially in the craniofacial and vertebral tissues, at E19.5. In addition, conditional deletion of *Integrin β1* in preosteoblasts using *Osx-Cre* transgenic mice resulted in viable mice that were normal at birth but displayed early defects in calvarial ossification, incisor eruption and growth.^87^*Osx-Cre; Integrin β1*^*fl/fl*^*mice* had reduced BMD, abnormal bone structure, and defects in mechanical properties. Although these defects persisted into adulthood, they became milder with age.

**Table 5:** Animal studies focused on Integrins in osteocytes

Targeted cells	Genotype	Baseline skeletal phenotype	Mechanical stimulation	Ref.
Global				
*Integrin β1*^*−/−*^		Embryonic lethality (dysfunctions of inner cell mass and collapsed blastocoeles).	n/a	^86^
*Integrin β3*^*−/−*^		No reported skeletal phenotype.	n/a	^90^
Mesenchymal condensation cells				
*Twist2-Cre; Integrin-β1fl/fl*		Die at birth; impaired skeletal development, especially in the craniofacial and vertebral tissues at E19.5 stage.	n/a	^87^
Preosteoblasts				
*Osx-Cre; Integrin-β1*^*fl/fl*^		Normal at birth but displayed early defects in calvarial ossification; lower bone mineral density and abnormal bone structure at adult stage; defects in mechanical properties.	n/a	^87,113^
Mature osteoblasts and osteocytes				
*OC-Cre; Integrin-β1*^*fl/fl*^		No effect on mineral density, biomechanics or fracture healing, just with some minor alterations of femur structure.	n/a	^87^
*Cola1(I)2.3-Cre; Integrin-β1*^*fl/fl*^		No observable skeletal phenotype at the proximal tibia, the distal femur, or lumbar vertebrae; reduced cell dendrites in cortical osteocytes.	Challenged with three consecutive days of cyclic ulna loading, a significant reduction in bone-formation rates was observed at the ulnar midshaft, compared with floxed *Integrin-β1*^*fl/fl*^ control mice	^88^
*Integrin β1-DN*^*OC*^		Reduced bone mass; increased cortical porosity in long bones, thinner flat bones in the skull; abnormal canaliculi structure in *β1-DN* mice, together with a higher staining for osteoclasts.	n/a	^89^

*Ref*. references, *n/a* not available, *OC* osteocalcin

Compared with its essential role in early bone development, the expression of *Integrin β1* in mature osteoblasts and osteocytes seems to be more related to osteocyte mechanosensation. Conditional deletion of *Integrin β1* in mature osteoblasts and osteocytes using *Oc-Cre* transgenic mice caused only minor alterations in femur structure without affecting BMD, biomechanics, or fracture healing.^87^ Similarly, deletion of *Integrin β1* using the 2.3-kb *Col1a1-Cre* caused no observable skeletal phenotype at the proximal tibiae, distal femurs, or lumbar vertebrae.^88^ However, transgenic mice overexpressing β1DN driven by the *Oc* promoter showed reduced bone mass, increased cortical porosity in the long bones and thinner flat bones in the skull.^89^ Detailed examination of these mice further showed an abnormal osteocyte canaliculi structure with increased osteoclast formation in the β1-DN mice. These inconsistent results may reflect the difference in KO and overexpression methodologies used to generate the transgenic mice, and β1DN overexpression have generated stronger effects on Integrin β1-associated signaling cascades. Interestingly, when *Cola1(I)-Cre; Integrin β1*^*fl/fl*^ mice, which had a normal skeletal appearance, were challenged with cyclic ulna loading for three consecutive days, a significantly reduced bone-formation rate at the ulnar midshaft compared with that in floxed *Integrin β1*^*fl/fl*^ control mice was observed.^88^ Together, these data suggest that Integrin β1, through its expression in MSCs and early osteoblast-lineage cells, plays a critical role in the regulation of embryogenesis and bone development. Moreover, the function of Integrin β1 in bone formation in mature osteoblasts and osteocytes is limited, but Integrin β1 plays important roles in osteocyte mechanobiology.

Compared with the number of in vivo studies focused on Integrin β1, fewer studies have examined Integrin β3 in bone and osteocytes (Table 5). Even though Integrin β3 has been shown specifically localize to osteocyte processes, where these cells sense external mechanical stimuli, no skeletal defects were reported in global *Integrin β3-d*eficienct mice.^90^ More investigations are required to demonstrate the possible roles of Integrin β3 in bone and bone adaptation to mechanical stimulation.

Taken together, both in vitro and in vivo data suggest the essential functions of Integrins in bone development and mechanical stimulation-associated bone homeostasis. The results demonstrate that Integrin β1 is important for early osteogenesis, such as that in bone marrow-derived MSCs and osteoblasts during cell differentiation. Since Integrin β3 is localized to the cell processes of osteocytes and directly contacts the canalicular wall, it could be important for osteocyte mechanotransduction. There could be functional redundancy between these two major β Integrins in osteocyte mechanotransduction, which could explain the normal skeletal phenotype in *Integrin β1* cKO transgenic mice. Therefore, there is an urgent need for more *in vivo* studies to show the involvement of Integrin β3 in bone development and bone mechanotransduction.

### GJs: connexins

In addition to cell-ECM connections through the FA complex, cells communicate with neighboring cells and the environment through GJs and hemichannels.^91^ GJs have been widely observed in various organs and systems, including epithelial tissue, eyes, ears, heart, nerve system, and skeletal system. Both GJs and hemichannels are composed of a protein known as connexin. A hexameric array of six connexin subunits gives rise to a connexon. Connexons can be composed of the same type of connexins (homomeric) or different types of connexins (heteromeric). GJs are composed of two juxtaposed connexons on the surfaces of adjacent cells, and unopposed connexons called hemichannels at the cell membrane act as direct conduits between the cytosol and extracellular environment.^92^ These intercellular and cell-extracellular environment channels allow the direct exchange of ions, nucleotides, small molecules and second messengers (those less than ~1.2 kDa in size, such as ATP, prostaglandin, and IP3).^91,92^

**Fig. 6:**
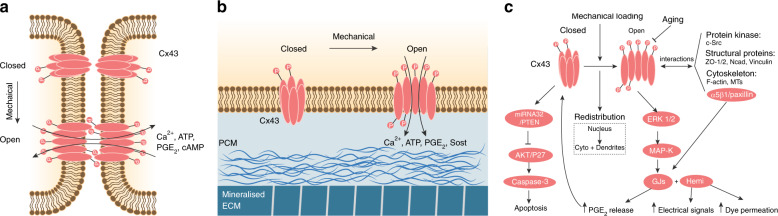
Osteocyte gap junctions and hemichannels in mechanobiology. **a** Illustration of osteocyte GJs in response to mechanical stimulation. A hexameric array of six connexin subunits gives rise to a connexon, and two juxtaposed connexons on the surfaces of adjacent cells form a GJ.^91,92^ When osteocytes experience mechanical stimulation, the Cx43 protein is phosphorylated, and the connexon is opened, allowing the exchange of several effectors, such as calcium, ATP, PGE_2_, and cAMP, between connecting cells. **b** Illustration of osteocyte hemichannels in response to mechanical stimulation. Unopposed connexons called hemichannels at the cell membrane act as direct conduits between the cytosol and extracellular environment.^91^**c** Signaling pathways involved in Cx43-based GJs and hemichannels during osteocyte mechanobiology

In the skeleton, GJs are present in all cell types and particularly abundant in osteoblasts and osteocytes.^92^ Osteoblasts and osteocytes express multiple types of connexins, including *Cx40*, *Cx43*, *Cx45*, *Cx46*, and *Cx37*, among which *Cx43* is a highly expressed GJ protein in bone.^92^ Cx43 and other connexins form a functional “3D syncytium” that connects different cells throughout the bone.^55^ Gap junctional intercellular communication (GJIC) orchestrates the formation and turnover of bone under physiological and pathological conditions.^91,93^ In particular, Cx43 regulates osteoblast formation, differentiation, survival and apoptosis. Cx43 also participates in the regulation of osteoclast formation and resorption ability. In osteocytes, Cx43-dependent GJIC and hemichannels contribute to the coordination of bone remodeling in response to anabolic factors and mechanical loading (Fig. 6).

Cx43 is vital for animal embryogenesis and bone development. Global deletion of *Cx43* in mice resulted in both neural crest cell defects and osteoblast dysfunctions, leading to animal death immediately after birth.^94^*Cx43*^*−/−*^ mice showed delayed intramembranous and endochondral ossification of the cranial vault and long bones during embryogenesis with skeletal abnormalities in the clavicles, ribs, vertebrae, and limbs. Cultured primary osteoblasts derived from *Cx43*^*−/−*^ mice exhibited decreased dye coupling compared with that of cells derived from wild-type (WT) control mice. Moreover, *Cx43* is involved in aging-related bone loss.^95^ Aged C57BL/6 mice displayed reduced *Cx43* expression in the bone, which was associated with increased osteocyte apoptosis, increased osteoclast number and enhanced bone resorption on the endocortical bone surface. Furthermore, mice carrying a G60S mutation in *Cx43* exhibited severe bone mass loss and decreased strength, highly similar to the symptoms of the human disease oculodentodigital dysplasia, which is caused by *Cx43* mutation.^91^ These results suggest the important role of Cx43 in bone development and homeostasis.

Unlike those with global deletion of *Cx43* in osteoblasts and osteocytes, mice with conditional deletion of *Cx43* in osteoblasts and osteocytes were viable but developed osteopenia. *Cx43* haploinsufficiency in osteoblasts (2.3 kb *Cola1(I)-Cre; Cx43*^*−/fl*^) led to normal mice at birth, but these mice developed reduced bone formation and defective osteoblast functions at six months of age.^96^ This osteopenic phenotype resulted from direct osteocyte loss. Similarly, deletion of *Cx43* in mature osteoblasts and osteocytes with the osteocalcin promoter (*Oc-Cre; Cx43*^*−/fl*^) increased osteocyte apoptosis in the cortical shell of lumbar vertebrae.^97^ TEM images further revealed the features of osteocyte apoptosis, including chromatin condensation, nuclear fragmentation, and even empty lacunae. Moreover, more osteoclasts were observed at the site of apoptotic osteocytes. Interestingly, bone-formation indexes on the periosteal surface were higher in *Oc-Cre; Cx43*^*−/fl*^ mice than in their *Cx43*^*−/fl*^ control littermates, whereas no difference was observed on the endocortical surface. Consistent with these results, deletion of *Cx43* in the osteocytes of *Dmp1-Cre; Cx43*^*fl/fl*^ mice resulted in increased osteocyte apoptosis and a higher prevalence of empty lacunae but did not affect bone mass.^97^ IHC data from bone sections indicated the reduced prevalence of Opg-expressing osteocytes in *Dmp1-Cre; Cx43*^*fl/fl*^ mice, but no change in the Rankl-positive osteocyte ratio was detected. Moreover, the loss of viable osteocytes in *Dmp1-Cre; Cx43*^*fl/fl*^ mice reduced local sclerostin levels, which further contributed to increased local bone formation. Together, these observations suggest that *Cx43* regulates osteocyte apoptosis and target protein expression.

In addition to the osteopenia developed by *Cola1(I)-Cre; Cx43*^*−/fl*^ mice, *Cx43* deficiency in osteoblasts attenuated the anabolic response to in vivo mechanical loading.^98^ After being subjected to a three-point bending protocol for two weeks, the loaded tibiae from *Cola1(I)-Cre; Cx43*^*−/fl*^ mice showed significantly reduced mineral apposition and bone-formation rates relative to those of control *Cx43*^*+/fl*^ mice. Moreover, these *Cola1(I)-Cre; Cx43*^*−/fl*^ mice needed ∼40% more force to generate the required endocortical strain. Similar to these defects during mechanical loading, mice with conditional deletion of *Cx43* in mature osteoblasts and osteocytes (*Oc-Cre; Cx43*^*fl/fl*^) failed to properly respond to mechanical unloading.^99^ At baseline, *Oc-Cre; Cx43*^*fl/fl*^ mice had an osteopenic phenotype in cortical bone but not in trabecular bone. When both *Oc-Cre; Cx43*^*fl/fl*^ and control mice were challenged with three weeks of mechanical unloading via HLU, the significant loss of cortical bone was observed. However, mechanical testing revealed the increased loss of bone strength and rigidity in *Oc-Cre; Cx43*^*fl/fl*^ mice compared with control mice after HLU. Moreover, *Oc-Cre; Cx43*^*fl/fl*^ mice did not experience HLU-induced bone loss in trabecular bone. Therefore, together, these data indicate that *Cx43* deficiency desensitizes bone to the effects of mechanical loading and unloading.

In addition to its role in mechanical stimulation, Cx43 in osteocytes plays a role in hormone stimulation. Even though conditional deletion of *Cx43* in osteocytes (*Dmp1-Cre; Cx43*^*fl/fl*)^ did not impact animal body weight or BMD, the mutant mice displayed different responses to anabolic PTH treatment.^100^ Intermittent PTH administration had similar effects in control and *Dmp1-Cre; Cx43*^*fl/fl*^ mice, as measured by the increase in bone mineral deposition and enhanced expression of osteoblast-related genes (*Alp, Runx2, Oc*, and *bone sialoprotein*). However, collagen fibers in *Dmp1-Cre; Cx43*^*fl/fl*^ mice failed to mature after PTH injection. In addition to its role in PTH signaling, Cx43 is involved in the estrogen pathway.^101^ When an ovariectomized (OVX) mouse model was developed to mimic estrogen deficiency, IHC results suggested that the OVX group has less staining for the Cx43 protein than the sham group. In this study, three types of mice with different genotypes were used: *WT* mice, *Cx43*^*R76W*^ mice (dominant-negative mutant in which only GJ channels were inhibited) and *Cx43*^*Δ130–136*^ mice (dominant-negative mutant in which both GJ channels and hemichannels were compromised). Compared with *WT* and *Cx43*^*R76W*^ mice, *Cx43*^*Δ130–136*^ mice had significantly decreased vertebral trabecular bone mass and increased apoptotic osteocytes. However, the osteoclast surface in trabecular and cortical bone after OVX was increased in *WT* and *Cx43*^*R76W*^ mice but not in *Cx43*^*Δ130–136*^ mice. These observations suggest that Cx43 in GJs and hemichannels may have different roles in regulating osteocyte and osteoclast activities and that intact Cx43-associated channels are essential for protection of the bone against catabolic effects resulting from estrogen deficiency.

Together, these in vivo observations demonstrate that Cx43 is not required for early osteogenesis but plays important roles in regulating anabolic responses in response to hormone treatment and mechanical stimulation in osteoblasts and osteoclasts (Table 6).

**Table 6:** Animal studies focused on Connexin-43 in osteocytes

Targeted cells	Genotype	Baseline skeletal phenotype	External stimulation		Ref.
			Mechanical	Chemical	
Global					
*Cx43*^*−/−*^		Animal die shortly after birth; delayed intramembranous and endochondral ossification during embryogenesis; skeletal abnormalities in clavicles, ribs, vertebrae and limbs.	n/a	n/a	^94^
*Cx43*^*R76W*^		n/a	n/a	Increased osteoclast surface after VOX in WT and *Cx43*^*R76W*^ mice, but not in *Cx43*^*Δ130–136*^ mice.	^101^
*Cx43*^*Δ130–136*^		n/a	n/a	*Cx43*^*Δ130–136*^ mice had significant decreased vertebral trabecular bone mass and increased apoptotic osteocytes in VOX model, compared with WT and *Cx43*^*R76W*^ mice.	^101^
Osteoblasts and osteocytes					
*Cola1(I) 2.3-Cre; Cx43*^*–/fl*^		Normal mice at birth; develop reduced bone formation and defective osteoblast functions at six-month; observed osteocyte loss.	Loaded tibia from CKO mice showed significantly lower mineral apposition rate and bone-formation rate; CKO mice needed ∼40% more force to generate the required endocortical strain.	n/a	^96^
*OC-Cre; Cx43*^*–/fl*^		Increased osteocyte apoptosis in the cortical shell of lumbar vertebrae; increased endocortical resorption, and periosteal bone formation; higher marrow cavity and total tissue areas measured at the femoral mid-diaphysis.	Failed to properly respond to mechanical unloading.	n/a	^97^
*OC-Cre; Cx43*^*fl/fl*^		Osteopenia phenotype in cortical bone, but not in trabecular bone.	Significant bone loss in cortical bone, but not cortical bone, were observed in both CKO mice in HLU experiments; mechanical testing revealed a greater loss of bone strength and rigidity for CKO mice after HLU.	n/a	^99^
Osteocytes					
*Dmp1-Cre; Cx43*^*fl/fl*^		Increased osteocyte apoptosis with higher prevalence of empty lacunae, but not affect bone mass; reduced prevalence of *Opg*-expression osteocytes in CKO mice, but no changes for *Rankl*-positive osteocyte ratio between groups; Reduced the sclerostin levels locally.	n/a	n/a	^97^
*Dmp1-Cre; Cx43*^*fl/fl*^		No difference in animal body weight and bone mineral density.	n/a	CKO mice failed to increase maturity of collagen fibers after PTH injection.	^100^

*Ref*. references, *n/a* not available, *OC* osteocalcin, *CKO* conditional knockout, *HLU* hindlimb unloading

In addition to in vivo studies, in vitro studies have revealed the detailed molecular contribution of Cx43 to osteocyte mechanotransduction (Fig. 6c). As observed in vivo, the major GJ protein expressed in MLO-Y4 cells is Cx43, and ~5% of total Cx43 is phosphorylated.^102^*Cx43*-silenced MLO-Y4 cells underwent spontaneous cell death through the AKT/P27/Caspase-3 pathway.^95^ Furthermore, fluid stress stimuli regulated Cx43 protein expression and subcellular distribution in MLO-Y4 cells.^102–104^ Both pulsating and steady fluid flow shear stress over MLO-Y4 cells redistributed the Cx43 protein from the perinuclear region (<2 μm around nuclei) into the cytoplasm and dendritic processes (>2 μm away from nuclei), which was associated with increased intercellular coupling.^102^ Cx43 protein expression was elevated 30 min after the application of stress but decreased at 24 h after stress. In a similar study, MLO-Y4 cells were exposed to 1 h of oscillating fluid flow, which increased the phosphoserine content of Cx43 by approximately twofold compared with that in cells without flow treatment.^104^ Moreover, oscillating fluid flow promoted the formation of new GJs between MLOY-4 cells without affecting dye transfer between established GJs, which was dependent on the ERK1/2-MAP kinase pathway.^104^

In addition to Cx43-mediated GJs, osteocytes use Cx43-dependent hemichannels in response to extracellular stimuli (Fig. 6b). When MLO-Y4 cells were plated at a low density in culture, Cx43 formed hemichannels instead of cell-cell contact GJs in individual cells.^105^ When these cells were subjected to fluid flow, the hemichannels of cells plated at a lower density released more PGE_2_ than those of cells plated at a higher density. Inhibitors of other channels, such as the purinergic receptor P2X7 and the prostaglandin transporter PGT, had no effect on PGE_2_ release. These data suggest that hemichannels regulate PGE_2_ release in osteocyte mechanotransduction.

Interestingly, Cx43 also participates in osteocyte autocrine effects. Conditioned medium obtained from fluid flow-treated MLO-Y4 cells increased Cx43 protein expression in static cultured MLO-Y4 cells.^103^ Treatment with purified PGE_2_ had an effect similar to that of fluid flow induction, suggesting that PGE_2_ is an autocrine effector responsible for *Cx43* expression. When PGE_2_ was depleted from the fluid flow conditioned medium, the stimulatory effect on GJs was partially but significantly decreased. These data suggest that Cx43 can regulate osteocyte autocrine effects under conditions of mechanical stimulation and that released PGE_2_ further enhances osteocyte *Cx43* expression in a positive feedback loop, eventually resulting in broader and more intense responses.

The function of Cx43 in osteocyte mechanobiology seems to be related to FAs, especially Integrins. MLO-Y4 cells cultured on a soft substrate expressed less *Cx43, Vinculin, Paxillin*, and *Fibronectin* than cells cultured on a stiff substrate.^106^ Immunoprecipitation (IP) results suggested a protein–protein interaction between Cx43 and Paxillin. Moreover, Cx43 was shown to colocalize with Integrins α5β1 in both MLO-Y4 cells and primary osteocytes through IF and IP experiments.^107^ Following chemical and mechanical activation of Integrins, MLO-Y4 cells were more permissive to dye diffusion via GJs. However, following siRNA-mediated knockdown of *Integrin α5* expression, the cells were less permissive to the dye. The same research group further showed that Integrins α5β1 directly interact with Cx43 through their C-termini, which promoted Cx43-mediated GJs and hemichannel opening in response to shear stress.^108^ This process is partially dependent on the PI3K pathway.^108^ Moreover, locally applied pN-level force-induced electrical coupling between MLO-Y4 cells required the release of ATP by the stimulated cells, and the junctional conductance increases with the number of junctional connections between cells.^59^

In addition to Integrins, GJIC and the activity of hemichannels, which are mediated through Cx43, are closely regulated by other cellular components.^109^ A number of Cx43-interacting proteins, including regulatory protein phosphatases and protein kinases (c-Src is the best studied), structural proteins (notably zona occludens-1/ZO-1, ZO-2, N-cadherins, Vinculin, etc.), and cytoskeletal proteins (MTs and actin), have been recently reported.^110,111^ These GJ-interacting proteins link Cx43-mediated channels to other subcellular functional compartments and may facilitate the additional roles of Cx43 in transcriptional and cytoskeletal regulation.

In summary, Cx43-mediated GJs and hemichannels play a crucial role in regulating bone homeostasis, especially in responses to hormone and mechanical stimulation.^112^ First, Cx43-mediated GJs can conduct chemical and mechanical signals among bone cells that translate both anabolic and catabolic responses from osteocytes into bone remodeling. Second, Cx43-mediated hemichannels contribute to autocrine or even endocrine effects. Third, Cx43 directly regulates osteocyte apoptosis, which further influences local osteoblast and osteoclast activity. Finally, Cx43 is in direct and indirect contact with FAs, structural proteins and protein kinases in osteocytes, which further facilitates bone homeostasis.

### Ion channels

**Fig. 7:**
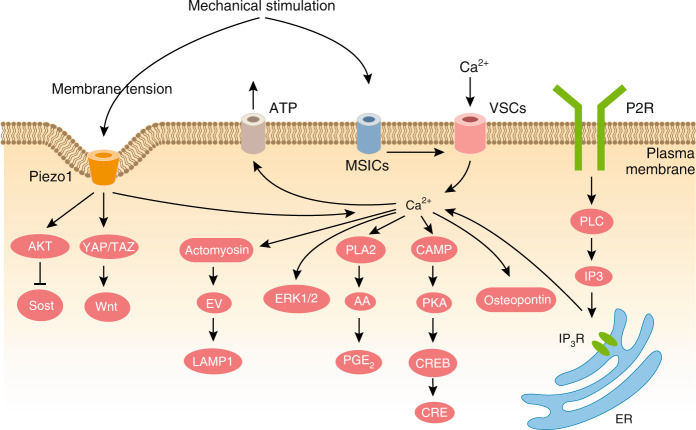
Illustration of ion channels involved in osteocyte mechanobiology. During osteocyte mechanotransduction, the earliest event that takes place is an increase in the intracellular Ca^2+^ concentration of the cells.^113^ This calcium mobilization process is first triggered by the activation of MSICs.^114^ Among all the MSICs, Piezo1 is a promising mechanogating ion channel in osteocyte mechanobiology. Piezo1 is a curved channel that is highly engaged with the cell membrane.^119^ Mechanical stimulation increases the osteocyte membrane tension, which further induces the opening of Piezo1 channels. Downstream effectors of Piezo1 channels include the Akt–Sost pathway,^124^ YAP/TAZ–Wnt pathway,^120^ and intracellular calcium signaling.^124^ Upon MSIC opening, ions are exchanged between the cytoplasm and extracellular environment. This process further changes the plasma membrane charge balance and induces the opening of VSCs.^125^ Interestingly, the calcium that undergoes flux induced by mechanical stimulation is derived from not only external fluid and medium but also sites of internal calcium storage, such as the endoplasmic reticulum.^128^ This calcium mobilization can activate downstream effectors, such as actomyosin, Erk1/2, PGE_2_, PAK, and osteopontin.^115^ Calcium mobilization also regulates ATP release in osteocytes upon mechanical stimulation.^113^

During cell mechanotransduction, the earliest event that takes place within 1 min of mechanical stimulation is an increase in the intracellular Ca^2+^ concentration in cells.^113^ In cultured bone cells, including osteocytes, osteoblasts and osteoclasts, with the results of both patch-clamp recording and calcium-sensitive fluorescent dye detection showed fast and mechanically stimulated calcium flux.^114,115^ This calcium mobilization process is first triggered by the activation of mechanical-sensitive ion channels (MSICs),^114^ which are opened by membrane tension changes from plasma membrane disruption under loading^116^ (Fig. 7). Ex vivo mechanical loading experiments showed that the blockade of MSICs, which are mainly stretch/strain-sensitive cation channels, with GdCl abolished PGI_2_ and NO production in osteocytes.^117^

Among all the MSICs, Piezo1 is a promising mechanogating ion channel for osteocyte mechanobiology. Discovery of the molecular structure of Piezo1 showed that Piezo1 is a large ion channel with a special and highly curved blade-like shape^118,119^ (Figs. 1 and 7). This curved channel is highly engaged with the cell membrane, amplifying the sensitivity of Piezo1 to changes in membrane tension.^119^ Moreover, *Piezo1* is expressed in osteoblasts and osteocytes and regulates mechanical load-dependent bone formation.^120,121^ Conditional deletion of *Piezo1* in osteoblasts and osteocytes with either *Oc-Cre* or *Dmp1-Cre* transgenic mice resulted in significantly reduced bone mass and impaired bone structure and bone strength.^120–122^ In vivo studies further showed that osteoblastic *Piezo1-deficient* mice were resistant to HLU-induced bone loss^122^ and that the loss of *Piezo1* in osteocytes compromised skeletal responses to mechanical loads.^123^ However, WT mice administered a Piezo1 agonist showed significantly increased bone mass, mimicking the mechanical loading effect.^120^ Furthermore, examination of human bone samples showed a negative correlation between *PIEZO1* expression and osteoporosis levels.^121^ In addition, in vitro cell culture studies suggested that *Piezo1* regulates osteocyte mechanobiology through several different pathways, including the Akt–Sost pathway^124^ and YAP/TAZ–Wnt pathway,^120^ and regulates intracellular calcium signals^124^ (Fig. 7).

Upon MSIC opening, ions, especially cations, are exchanged between the cytoplasmic and extracellular environments. This process further changes the plasma membrane charge balance and induces the opening of voltage-sensitive calcium channels (VSCs).^125^ In osteocytes, T- and L-type VSCs are coexpressed and both contribute to mechanically induced calcium flux. For example, in osteocyte-like MLO-Y4 cells, both T- and L-type VSCs are expressed and accelerate ATP release and Erk1/2 activation.^126,127^ Moreover, during in vivo ulna loading, blockade of L-type VSCs with a specific antagonist (nifedipine or verapamil) in loading rats before experiments reduced loading-induced bone formation by 50%–60%.^113^ With the sequential opening of MSICs and VSCs, calcium flux takes place in mechanically activated osteocytes.

Interestingly, the calcium that undergoes flux induced by mechanical stimulation is derived from not only external fluid and the medium but also stored internal calcium, such as that in the endoplasmic reticulum (ER). On the one hand, when cells were cultured in medium lacking calcium, bone cells lost the ability to activate calcium flux upon fluid flow.^128^ On the other hand, blockade of ion channels with inhibitors in both cell culture and animal models only partially inhibited flow responses.^114,129^ In addition, cultured cells treated with thapsigargin (a drug that depletes intracellular calcium stores) significantly reduced the occurrence of calcium peaks in response to fluid flow.^128^ These observations suggest that calcium release from intracellular stores (such as the ER) also participates in the calcium response to flow.

These results further show that calcium mobilization in osteocytes is a quantitative response to mechanical loading. Ex vivo loading experiments revealed that the ratio of responding osteocytes was controlled by the loading frequency and magnitude, but the Ca^2+^ intensity within each osteocyte remained consistent during the response.^130^ Moreover, this calcium mobilization activated expression of the downstream signal molecules Erk1/2, PGE_2_, PAK, and osteopontin^115^ (Fig. 7). Moreover, calcium mobilization also regulated ATP release in osteocytes upon mechanical stimulation. In rat ulnar loading experiments, pretreatment with two L-type VSC antagonists (nifedipine and verapamil) reduced load-induced bone formation and ATP signaling.^113^ Taken together, these results show that MSICs and VSCs actively participate in osteocyte mechanotransduction. Calcium, the release of which is a very early event in osteocyte mechanotransduction, serves as a powerful second messenger, providing essential information for mechanical responses and participating in downstream regulation.

### ECMs: glycocalyx

The primary ECM components in bone are organic collagen and inorganic hydroxyapatite.^131^ Among these components, the collagen network contributes to the toughness of bone and its resilience to fracture but has little effect on the stiffness of bone.^132^ During osteocyte maturation, interaction between collagen networks, osteoblast orientation and the lacunae structure has been reported.^133^ The results of a detailed examination of equine, ovine, and murine bones suggested that osteoblasts are aligned with the highly oriented collagen matrix.^133^ Moreover, newly synthesized collagen matrix from osteoblasts adopts a preferential orientation in the direction of the cell.^134^ As a result of the interaction between the matrix and osteogenic differentiation, osteocytes are embedded in a well-polarized environment.^135^ Moreover, during development, tension, compression, and shear forces from animal activities over time define the main axes of osteocytes and osteocyte lacunae, which are highly oriented along the force direction.^131,136^ These observations suggest interactive communication between the ECM environment and osteocytes during development, especially during mechanical stimulation.

Even though osteocytes are embedded in the LCS, which allows fluid flow and nutrient exchange for these cells, the space between the LCS wall and the osteocyte plasma membrane is not empty. As discussed, and as presented in Fig. 1, high-resolution TEM and SEM studies revealed PCM and collagen hillocks between the osteocyte cell body and lacunae and between cell processes and canaliculi. In these spaces, together, the ECM components proteoglycans, glycoproteins, and hyaluronic acid form a mesh of PCM, called the glycocalyx.^137^ The glycocalyx is required to link the osteocyte membrane to LCS walls and serves to sense and transduce mechanical signals from the ECM or fluid flow to osteocytes.^137^

The large, monomeric heparan sulfate proteoglycan protein Perlecan/Shpg2 has been reported to be a suitable candidate for the functions listed above.^138^ First, IF and immunogold detection of *Perlecan* revealed its localization in the pericellular space of the osteocyte LCS in cortical bone.^139^ Second, atomic force microscopy examination of purified full-length human PERLECAN showed that this protein has an end-to-end length of 170 ± 20 nm and a diameter of 2–4 nm, allowing it to function as a tethering element that connects the osteocyte cell body to the bone matrix.^140^ Moreover, the Perlecan protein is strong but elastic (elastic constant of 890 pN, Young’s modulus of 71 MPa), which allows the protein to withstand any drag forces from physiological fluid flow.^140^ Third, Perlecan is essential for the integrity of the osteocyte LCS.^139^ Global deletion of *Perlecan* in mice resulted in reductions in the total canalicular area, canalicular density and number of transverse tethering elements in the canaliculus.^79^ Fourth, *Perlecan*-deficient mice exhibited higher solute transport and higher shear stress in the bone.^141^ Fifth, when these mice were subjected to mechanical tibial loading, loading-induced anabolic bone formation was abolished, but this was not observed in control mice.^141^ Real-time calcium recording further showed that *Perlecan*-deficient mice experienced decreases in the overall Ca^2+^ response rate, calcium peaks, peak magnitude, and recovery speed to baseline.^142^

In summary, ECM components, especially the glycocalyx in the pericellular space, actively contribute to osteocyte mechanotransduction. These glycocalyx molecules act as a tether or ligand for osteocyte membrane receptors, conveying mechanical signals due to either ECM deformation or fluid shear to the osteocyte surface. All of these important functions make the glycocalyx in the ECM an essential osteocyte mechanosensor during force adaptation.

## Signaling pathways that regulate osteocyte mechanobiology

**Fig. 8:**
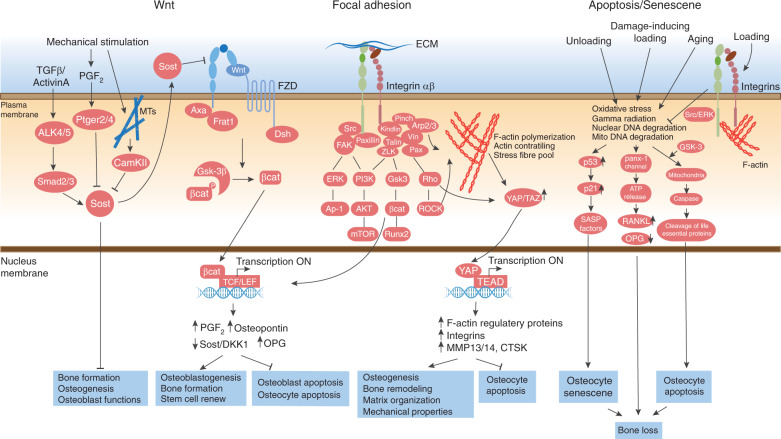
Signaling pathways involved in osteocyte mechanobiology. The Wnt/β-Catenin pathway mechanistically, the canonical Wnt/β-Catenin pathway is activated through the binding of Wnt ligands to a coreceptor complex consisting of Lrp5 or Lrp6 and FZD.^152,153^ This binding further activates the intercellular effector Dsh by FZD-mediated phosphorylation. Activated Dsh leads to the phosphorylation of Gsk-3β, which inhibits free β-Catenin in the cytosol by phosphorylating β-Catenin at multiple serine/threonine sites. Once Gsk-3β is phosphorylated by Dsh, it releases captured β-Catenin. As a result, free β-Catenin is translocated to nuclei, where it binds the coeffectors Tcf and Lef, inducing downstream gene transcription. Downstream effects of β-Catenin include the expression of Wnt target genes^154^ and secretory proteins (Opg, Osteopontin)^157,161^ and load-induced PGE_2_ secretion.^164^ Sclerostin antagonizes Wnt signaling through its competitive binding to Lrp5 and Lrp6 at their first two YWTD-EGF repeat domains.^181^ Mechanical stimulation can suppress *Sost* expression through both Peger2/4 and the MT pathway. In addition, the Tgfβ-Smad2/3 pathway can enhance sclerostin expression. As a result, during the osteocyte mechanotransduction process, the Wnt/β-Catenin pathway enhances osteoblastogenesis and bone formation; however, sclerostin negatively regulates the Wnt/β-Catenin pathway. Focal adhesion As the central proteins in the FA complex, Integrins, especially Integrin β subunits, are essential for bone development and osteocyte mechanotransduction. The “Integrin adhesome” is a network of 156 proteins in the FA complex.^166^ In the FA complex, Kindlin-2, Talin, and other structural proteins are directly linked to the cytoplasmic tail of the Integrin β subunit, which further connects with the Pinch, Paxillin, Vinculin, and Arp2/3 proteins.^23,79^ This Integrin adhesome complex links the ECM and F-actin cytoskeleton and enhances the activation of downstream pathways, such as the Erk, PI3K, Gsk3, and Rho pathways. Upon F-actin cytoskeleton polymerization, YAP/TAZ coordinate signals from Rho GTPase and tension of the actomyosin cytoskeleton, initiate downstream target gene expression, and finally enhance osteogenesis and bone remodeling and inhibit osteocyte apoptosis. Apoptosis/senescence osteocyte apoptosis, a form of programmed cell death, and senescence, a death-resistant cell fate program, are common features of aging bone tissue. Appropriate mechanical stimulation prevents osteocyte apoptosis, whereas aging, damage-inducing loading and disuse induce osteocyte apoptosis^174^ and senescence^177^ through several different pathways. In contrast, mechanical stimulation induces Src/Erk activation through Integrin and the cytoskeleton in osteocytes, inhibits apoptotic and senescence-related pathways and supports osteocyte survival.^172^

During skeletal development, multiple classical signaling pathways have been shown to play indispensable roles in bone formation through both genetic modification of experimental animals and human genomic studies. These classical pathways include the Notch, Hedgehog, Bmp, and FGFR signaling pathways.^143^ These pathways exhibit some common features. Receptors on the plasma membrane are activated through direct binding to specific ligands, leading to the activation of sequential signaling cascades and expression of downstream target genes.^144–147^ All of these pathways are involved in the regulation of bone-formation processes, such as osteoblast differentiation and mineralization and limb patterning. Interestingly, Notch signaling has dual effects on osteogenesis and osteoclastogenesis.^148^ On the one hand, Notch enhances osteoblast mineralization, osteogenic differentiation and thereby bone formation; on the other hand, Notch also facilitates bone resorption. In addition to traditional pathways in bone development, several classical and new pathways that regulate bone homeostasis during osteocyte mechanotransduction have emerged (Fig. 8).

### Wnt/β-Catenin

The Wnt signaling pathway, the major regulator of the transition of stem cells into mature osteoblasts, has been proven to be a key regulator of bone mass. Mutations of *WNT1* in humans cause early-onset osteoporosis and osteogenesis imperfecta.^149,150^ A comprehensive study of transgenic mice carrying mutations in the Wnt pathway showed the association between experimental phenotypes and clinical observations in human bone.^151^

Mechanistically, the canonical Wnt/β-Catenin pathway is activated through the binding of Wnt ligands to a coreceptor complex (Fig. 8) consisting of Lrp5 or Lrp6 and the frizzled family member FZD.^152,153^ This binding further activates the intercellular effector Dishevelled (Dsh) by FZD-mediated phosphorylation. Dsh activation leads to the phosphorylation of Glycogen Synthase Kinase-3β (Gsk-3β), which inhibits free β-Catenin in the cytosol by phosphorylating β-Catenin at multiple serine/threonine sites. Once GSK-3β is phosphorylated by Dsh, it releases captured β-Catenin. As a result, free β-catenin is translocated to nuclei, where it binds its coeffectors Tcf and Lef, inducing downstream gene transcription. In addition to its functions in bone development, the Wnt pathway is an important osteocyte mechanotransduction pathway. For example, during cyclic compression loading in WT mice, significant expression of the Wnt pathway-related genes encoding Wnt10B, SFRP1, Cyclin D1, FzD2, WISP2, and Cx43 was induced.^154^

As a major receptor in the Wnt pathway, Lrp5 has been shown to be highly important in osteocyte mechanotransduction. In humans, loss-of-function mutations in *LRP5* are tightly associated with osteoporosis–pseudoglioma syndrome, which is characterized by low BMD and skeletal fragility,^155^ and gain-of-function mutations in *LRP5* lead to a high bone mass phenotype.^156^ Experimental transgenic *Lrp5-null (Lrp5*^*−/−*^*)* mice had significantly reduced bone mass and strength compared with those in control mice.^157^ However, mice expressing constitutively activated mutant *Lrp5* (*Lrp5*^*G171V*^) displayed a high bone mass phenotype with stronger mechanical properties, even those with a heterozygous background.^158^ When challenged with cyclic mechanical loading or disuse-associated mechanical unloading, *Lrp5-null* mice showed reduced osteogenic responses after loading^157,159^ and a greater loss of cancellous bone after disuse.^159^

The Wnt effector β-Catenin is also involved in osteocyte mechanical responses. Deletion of *β-Catenin* in osteocytes (*Dmp1-Cre; β-Cat*^*fl/fl*^) resulted in reduced skeletal mass.^160^ This low bone mass phenotype seemed to be related to an increased osteoclast number, but normal osteoblast function and osteocyte density were observed.^161^ In these mice, the anabolic effects of mechanical force loading were compromised.^160^ Moreover, β-Catenin may influence osteocyte mechanotransduction in a dose- and sex-related manner. Studies using heterozygous mice in which one copy of *β-Catenin* was deleted (*Dmp1-Cre; β-Cat*^*fl/+*^) showed opposite results. Bonewald et al. found that 5-month-old male, but not female, mice had significantly a reduced trabecular bone volume compared with that of control mice.^162^ However, Johnson et al. found that female, but not male, heterozygous mice showed a reduced trabecular bone volume and increased trabecular bone separation compared with those in their sex-matched controls.^163^ When these heterozygous mice were subjected to HLU experiments, female, but not male, mice displayed greater trabecular bone loss than controls.^162^ When these heterozygous mice were subjected to external loading experiments, neither male nor female heterozygous mice exhibited a significant increase in new cortical bone formation.^163^

Together, these data suggest that mechanical stimulation activates the canonical Wnt/β-catenin pathway and thereby influences osteocyte activity in load-induced bone formation. The downstream effects of *β-Catenin* include the expression of *Wnt* target genes^154^ and secretory proteins (Opg, Osteopontin),^157,161^ as well as load-induced PGE_2_ secretion.^164^

### FAs

Sensing of the external environment and stimulation by osteocytes is largely dependent on the connects between cells and the ECM–Integrin-based FAs. As discussed above (Fig. 3 and Table 5), Integrins, especially Integrin β subunits, are essential for bone development and osteocyte mechanotransduction. Interestingly, Integrins act not only as a physical connector of cells and ECM but also as a signal center for both “outside-in” and “inside-out” FA signaling.^23,82,165^ For “outside-in” signals, Integrins transmit external signals, including chemical and physical signals, into the cell and provide information regarding cellular location, microenvironment, and adhesive state. These signals activate internal signaling pathways that determine cell migration, survival and differentiation. In addition, “inside-out” signals from the cytoplasmic tails of Integrins influence the conformational changes of their extracellular domains and further regulate the affinity for extracellular ligands. With this bidirectional transmission between the intracellular environment and intercellular status, Integrins carry out dynamic, spatial, and temporal regulation in osteocytes (Fig. 8).

From a large literature study, an “Integrin adhesome” composing a network of 156 proteins was proposed to be associated with FAs.^166^ In addition to Integrin subunits, several FA-associated proteins have been proven to have essential roles in regulating bone development (Fig. 4). For example, Kindlin-2, a direct β1 Integrin- and β3 Integrin-binding protein, is essential for both bone development and osteocyte functions.^167,168^ Deletion of *Kindlin-2* in limb MSCs with *Prx1-Cre* caused neonatal lethality, chondrodysplasia, and loss of the skull vault by inhibiting *Sox9* expression and TGF-β signaling.^167^ Conditional deletion of *Kindlin-2* in osteocytes using a 10-kb *Dmp1-C*re transgene greatly altered the bone microenvironment and bone remodeling, resulting in a severe osteopenic phenotype.^168^ The results from this study demonstrated that Kindlin-2 controls the expression of sclerostin and Rankl in osteocytes and thereby bone remodeling. Another FA-binding protein, Pinch, is also involved in osteogenesis and osteocyte mechanosensation. Global *Pinch2* deletion and conditional *Pinch1* deletion in osteocytes resulted in significant bone loss in mice, whereas single deletion of one isoform of *Pinch* did not cause any marked skeletal phenotypes.^169^ The loss of both *Pinch* isoforms in osteocytes caused apoptotic death in cortical osteocytes with enhanced sclerostin detection in bone sections.^170^ Moreover, mice in which both *Pinch* isoforms were deleted from osteocytes showed reduced anabolic bone formation under mechanical loading conditions.^169^ Moreover, deletion of the FA signaling protein FA kinase in Osx-expressing cells also caused a low bone mass phenotype, resulting from compromised osteogenic differentiation.^171^

Taken together, these results show that the Integrin-centered FA signaling pathway is essential for skeletogenesis and tightly associated with bone mechanobiology. More studies focused on individual components of the adhesome are required, and osteocyte mechanobiology should be studied in more detail.

### Apoptosis and senescence

Osteocyte apoptosis, a form of programmed cell death, and senescence, a death-resistance cell fate program, are common features of aging bone tissue (Fig. 8). Both in vitro and in vivo studies have shown that appropriate mechanical stimulation prevents osteocyte apoptosis. The results from in vitro culture studies further suggested that mechanical stimulation induces Erk activation through Integrin and the cytoskeleton in osteocytes and supports osteocyte survival.^172^ However, damage-inducing loading induced osteocyte apoptosis,^173^ which is similar to the effects of disuse.^174^ In microcracks generated in overloading experiments, osteocyte apoptosis was found to occur adjacent to microdamage, accompanied by high *Bax* expression and TUNEL signaling.^175^ These apoptotic osteocytes further induced *Rankl* expression and osteoclastogenesis, which resulted in increased bone resorption and bone mass loss.^176^ Interestingly, in experimental animals, treatment with an apoptosis inhibitor (the pancaspase inhibitor Q-VD-OPh) rescued osteocyte cell loss and bone mass reduction during HLU and fatigue loading experiments.^173,174^

Compared with osteocyte apoptosis, less is known about senescence. Both apoptosis and senescence appear to be tightly associated with age-related bone loss through two independent pathways.^177^ Senescence is a cell fate program during which death-resistant cells respond to DNA damage to prevent the replication of DNA mutations. Senescent cells can release various cytokines, known as the proinflammatory secretome or senescence-associated secretory phenotype, causing significant damage to the surrounding tissues.^178^ Studies focused on endurance and resistance exercises suggested the beneficial effect of exercise on osteocyte viability, potentially through preserved mitochondrial function and the inhibition of osteocyte senescence.^177^ More evidence to illustrate the relationship between mechanical stimulation and osteocyte senescence is required and could provide useful insights into antiresorptive therapy by reduced osteocyte senescence and osteocyte apoptosis.^179^

### Other mechano-related pathways

#### Sclerostin

Sclerostin, an antagonist of Wnt//β-Catenin signaling, is widely known as an inhibitor of bone formation. In osteocytes, sclerostin is detected in the cell body as well as cell processes.^180^ Sclerostin antagonizes Wnt signaling through its competitive binding to Lrp5 and Lrp6 at their first two YWTD-EGF repeat domains.^181^ This binding disrupts Wnt-induced Frizzled-LRP complex formation and thus influences activation of the downstream effector β-Catenin^182^ (Fig. 8). In humans, genetic mutations in the *SOST* gene, which encodes SCLEROSTIN, cause sclerosteosis, an autosomal recessive disease characterized by high bone mass and bone tissue overgrowth.^183^ Similar to the results of human genetic studies, *Sost* deletion in mice resulted in a high bone mass throughout life, and these mice exhibited fewer apoptotic osteocytes and apoptotic osteoblasts in their bone tissue with enhanced Wnt//β-Catenin signaling.^184^

Most importantly, Sclerostin is involved in osteocyte mechanotransduction through its antagonistic effects on the Wnt//β-Catenin pathway and potentially other pathways. In WT mice, mechanical ulna loading reduced the level of *Sost* transcription, while HLU increased Sclerostin expression.^25^ These data are consistent with the increased circulating SCLEROSTIN level observed in humans during prolonged bed rest and immobilization.^43^*Sost-null* mice failed to sense unloading and displayed resistance to any bone loss.^184^ In addition to the antagonistic effects of Sclerostin on Wnt//β-Catenin signaling, in vitro culture experiments suggest that Sclerostin is involved in osteocyte Rankl and Opg expression,^43^ calcium flux,^53^ and oxygen sensing^185^ during FSS induction. Together, these data demonstrate that Sclerostin participates in osteocyte mechanobiology through Wnt signaling and potentially other pathways.

### YAP/TAZ

Yes-associated protein (YAP) and transcriptional coactivator with PDZ-binding motif (TAZ, also known as WWTR1) are two proto-oncogene proteins that are widely known as mechanosensors and mechanotransducers in various cell types.^186^ A pioneering work by Dupont et al. demonstrated YAP/TAZ translocation from the cytoplasm to the nucleus in MSC differentiation, a process that is decided by ECM stiffness and physical constraints.^187^ Moreover, in this process, YAP/TAZ acted as sensors and mediators of mechanical cues, coordinated with signals from Rho GTPase and tension of the actomyosin cytoskeleton, and finally contributed to cell differentiation^187^ (Fig. 8). Importantly, YAP/TAZ could respond to a complex physical microenvironment and mechanical cues, ranging from ECM properties, cell geometry, cell density, and cell polarity to shear stress.^188^ The overall consequences of YAP/TAZ activity during cellular mechanobiology include organogenesis, tissue homeostasis and multiple diseases, such as fibrosis, pulmonary hypertension, inflammation, muscular dystrophy, and cancer.^188,189^

In skeletal tissue, YAP/TAZ have been detected throughout osteolineage cell differentiation.^190^ In MSCs, mechanical niches trigger YAP/TAZ nuclear translocation, contributing to osteoblastogenesis and bone formation.^191^ In skeletal-lineage cells, deletion of *YAP/TAZ* using *Osx-Cre* caused an osteogenesis imperfecta-like phenotype, the severity of which was dependent on allele dose.^192^ Interestingly, a greater osteopenia phenotype was observed with homozygous *TAZ* deletion than with homozygous *YAP* deletion.^192^ In experimental animals, *YAP/TAZ* deletion reduced bone mass and bone material properties, which was associated with impaired collagen content and organization. In mature osteoblasts and osteocytes, *YAP/TAZ* increased osteoblast number and accelerated bone formation. Osteocyte-specific deletion of *YAP/TAZ* with *Dmp1-Cre* resulted in low bone mass, impaired bone mechanical properties, and disorganized collagen fibers.^193^ Furthermore, *YAP/TAZ* deletion in osteocytes reduced expression of the matrix proteases *Mmp13*, *Mmp14* and *Ctsk*, which resulted in reduced canalicular network density, length and branching.^193^ Together, these data suggest the important role of *YAP/TAZ* in osteogenesis and bone formation. Great effort is needed to determine the mechanism(s) of nuclear YAP/TAZ translocation during the mechanical stimulation of osteocytes.

## Conclusions and perspectives

With rapidly increasing attention on osteocyte mechanobiology, increasingly more players in mechanosensation and mechanotransduction are being discovered. A better understanding of the details of osteocyte mechanobiology will provide promising treatments for disease-, disuse-, and age-related bone loss. These treatments could be science-based advice regarding daily activities, noninvasive machine-based mechanical stimulation, or simply small molecules or peptides that regulate important players in osteocyte mechanobiology. One exciting example is the approval of romosozumab, a Sclerostin monoclonal antibody drug, for the clinical treatment of osteogenesis imperfecta and low BMD in postmenopausal women and patients with a high risk of fracture.^194^

The importance of osteocyte mechanobiology is related to not only bone health but also the homeostasis of other organs. On the one hand, as important endocrine cells, osteocytes can regulate the functions of multiple organs, such as muscle growth, memory in the brain, and fertility in the testis.^7^ Meanwhile, mechanical stimulation enhances and regulates osteocyte secretion activity.^1,4,6,10^ As a result, osteocyte mechanobiology participates in several different pathological conditions, such as OA,^195^ inflammation,^196,197^ bone metastases,^198,199^ and aging.^200,201^ For instance, unbalanced chronic overloading on one side of OA joints results in continuous thinning of the cartilage, bone attrition, and sclerosis.^202^ Osteocytes derived from OA patients have a rounded morphology with reduced Integrin β3 expression.^195,203^ Moreover, decellularized matrices from OA patients showed abnormal matrix components^195^ and increased stiffness.^204^ On the other hand, bone tissue is also a target for other organs. For example, adipocyte-specific *Kindlin-2* deletion caused a severe lipodystrophy phenotype.^205^ These transgenic mice had a high bone mass phenotype, which could have been the result of reduced leptin production from fat tissue in the animals, affecting bone metabolism.^205,206^

These results suggest interorgan communication between bone and distal organs, which indicates that the mechanotransductive properties and abilities of osteocytes can influence multiple cell types from different organs, including the surrounding bone cells and distal cells. Moreover, changes in possible ECM components and the morphology of osteocytes due to the influence of other organs can also affect osteocyte mechanobiology. Therefore, osteocytes residing in bone and other cells from various organs form a continuous loop that regulates whole-body homeostasis.

From the perspective of osteocytes, how these cells coordinate different mechanosensors and pathways in a complex stimulating environment, such as during daily physical activities, such as running, walking, and jogging, remains intriguing. Complex stimulation triggers more than one mechanosensor and activates more than one pathway, necessitating interactions between different players in the process. Therefore, considering osteocyte mechanobiology in a systematic way is a future direction for both basic research and potential clinical utilization. Moreover, osteocytes are not the only mechanosensitive cells in bone. In addition to these terminally differentiated cells, at least three different bone cell types, i.e., bone-resorbing osteoclasts, bone-forming osteoblasts, and osteoprogenitors, show the ability to sense and respond to biophysical signals.^18^ It is important to dissect these different cell types, different force origins, and the properties of different types of stimulation in the process to speculate on the difference between the responses of various cell types to complex physical stimuli and understand the possible universal roles of bone mechanobiology.
